# Distinct Solubilization Mechanisms of Medroxyprogesterone in Gemini Surfactant Micelles: A Comparative Study with Progesterone

**DOI:** 10.3390/molecules29204945

**Published:** 2024-10-19

**Authors:** Hiromichi Nakahara, Kazutaka Koga, Keisuke Matsuoka

**Affiliations:** 1 Department of Industrial Pharmacy, Faculty of Pharmaceutical Sciences, Daiichi University of Pharmacy, 22-1 Tamagawa-cho, Minami-ku, Fukuoka 815-8511, Japan; 2 Department of Kampo Pharmacy, Faculty of Pharmaceutical Sciences, Daiichi University of Pharmacy, 22-1 Tamagawa-cho, Minami-ku, Fukuoka 815-8511, Japan; k-koga@daiichi-cps.ac.jp; 3 Laboratory of Chemistry, Faculty of Education, Saitama University, 255 Shimo-Okubo, Sakura-ku, Saitama 338-8570, Japan; matsuokakei@mail.saitama-u.ac.jp

**Keywords:** gemini surfactant, solubilization, medroxyprogesterone, micelle, 2D ROESY, machine learning

## Abstract

The solubilization behavior of medroxyprogesterone (MP) within gemini surfactant micelles (14-6-14,2Br^−^) was investigated and compared with that of progesterone to uncover distinct solubilization mechanisms. We employed ^1^H-NMR and 2D ROESY spectroscopy to elucidate the spatial positioning of MP within the micelle, revealing that MP integrates more deeply into the micellar core. This behavior is linked to the unique structural features of MP, particularly its 17β-acetyl group, which promotes enhanced interactions with the hydrophobic regions of the micelle, while the 6α-methyl group interacts with the hydrophilic regions of the micelle. The 2D ROESY correlations specifically highlighted interactions between the hydrophobic chains of the surfactant and two protons of MP, H22 and H19. Complementary machine learning and electron density analyses supported these spectroscopic findings, underscoring the pivotal role of the molecular characteristics of MP in its solubilization behavior. These insights into the solubilization dynamics of MP not only advance our understanding of hydrophobic compound incorporation in gemini surfactant micelles but also indicate the potential of 14-6-14,2Br^−^ micelles for diverse drug delivery applications.

## 1. Introduction

Surfactant micelles are valuable for their ability to solubilize hydrophobic organic compounds, a property with diverse applications in industries such as medicine, pharmaceutics, and environmental science [1,2]. The efficiency of solubilization is a critical factor in the selection of an optimal surfactant, particularly as there is a growing emphasis on minimizing surfactant usage in practical applications. Nonionic surfactants, known for their relatively low critical micelle concentration (CMC) compared with conventional ionic surfactants, have been extensively studied for their potential as solubilizing agents [3,4]. However, recent research has also explored the solubilization capabilities of other surfactant types such as those of natural origin and hydrophobically modified polyelectrolytes, which have shown promising results [5,6,7]. In the context of drug delivery systems, the solubilization of hydrophobic bioactive compounds presents a significant challenge. Compounds such as sex hormones exhibit poor water solubility, which can severely limit their bioavailability and therapeutic efficacy. This issue underscores the need for advanced solubilization techniques that can enhance the solubility of such compounds, thereby improving their absorption and effectiveness in pharmaceutical applications.

Recent advances in the solubilization of hydrophobic drugs using micellar systems have significantly impacted the pharmaceutical industry, particularly in enhancing the bioavailability of poorly water-soluble compounds [8]. For example, the use of micelles in the solubilization of antidiabetic drugs and naturally derived compounds like curcumin has been shown to increase their bioavailability, leading to more effective drug formulations [9,10]. Moreover, mixed micellar systems have emerged as superior alternatives to single micelle systems, providing higher drug solubility, which is crucial for the development of viable therapeutic agents [11]. Despite these developments, the solubilization of highly hydrophobic bioactive compounds, including sex hormones, remains a challenging area. The inherent low aqueous solubility of these compounds not only limits their bioavailability but also poses significant hurdles in their integration into drug delivery systems. Conventional methods often struggle to maintain a stable therapeutic concentration of these drugs within the body, which is critical for achieving desired clinical outcomes. This issue is particularly acute for hormonal therapies, where precise and consistent dosing is essential for efficacy.

Gemini surfactants have recently attracted interest due to their unique structural characteristics, which enhance their ability to solubilize hydrophobic substances [12,13,14]. Characterized by two hydrophilic headgroups and a hydrophobic spacer, these surfactants form micelles with distinct properties, including a lower CMC and a higher micelle aggregation number compared with conventional surfactants. These properties make gemini surfactants particularly suitable for encapsulating hydrophobic molecules within the micellar core, thereby stabilizing them in aqueous environments [15,16,17]. Previous studies have demonstrated the ability of the gemini surfactant 14-6-14,2Br^−^ to effectively solubilize progesterone without significantly disrupting the micellar structure, suggesting a promising role for these surfactants in drug delivery applications [14].

Building on our previous research with progesterone, this study focuses on the solubilization behavior of medroxyprogesterone (MP) within the micelles of the gemini surfactant 14-6-14,2Br^−^. MP not only shares structural similarities with progesterone but also exhibits distinct biological activities. For example, when combined with human menopausal gonadotropin (hMG), MP influences ovarian follicular development by modulating hormonal levels, reducing luteinizing hormone (LH), estradiol (E2), and progesterone and activating the PI3K/Akt/mTOR signaling pathway, which is essential for follicular growth and reproductive functions [18]. Additionally, MP has significant metabolic effects on various tissues such as the breast, cardiovascular system, brain, and bone, highlighting its broader impact on body metabolism during hormone therapy in menopausal women [19]. Notably, unlike natural progesterone, MP does not stimulate the production of brain-derived neurotrophic factor (BDNF), crucial for neuroprotection and neural health, suggesting that different progestogens may lead to varying therapeutic outcomes, especially in menopausal conditions [20]. MP’s 17β-acetyl group plays a pivotal role in its deeper incorporation into the micellar core. This deeper integration is hypothesized to significantly influence the stability and solubilization efficiency of MP in comparison to progesterone, which tends to localize more at the micelle surface. Understanding these differences is critical for optimizing micellar drug delivery systems, particularly for hydrophobic compounds like steroid hormones. By utilizing advanced NMR techniques, including 2D ROESY spectroscopy, we aimed to elucidate the specific interactions between MP and the gemini surfactant, with a particular focus on the spatial positioning of MP within the micelle. This investigation is crucial for understanding how the unique structural features of MP affect its incorporation into the micellar structure compared with progesterone. Understanding these interactions is essential to optimize the use of gemini surfactants in drug delivery systems, where the solubilization efficiency and the precise positioning of the drug within the micelle can significantly impact its bioavailability and therapeutic efficacy [21,22,23,24]. Through a detailed analysis of NMR spectra, a structural fingerprint based on machine learning predictions, and an electron density map of MP, this study seeks to provide deeper insights into the molecular dynamics of MP solubilization within gemini surfactant micelles. The findings from this study are expected to contribute to the development of more effective micellar drug delivery systems, offering strategies to improve the solubilization of hydrophobic bioactive compounds and enhance their therapeutic potential.

## 2. Results and Discussion

### 2.1. ^1^H-NMR Spectra of 14-6-14,2Br^−^ and MP

**Figure 1:**
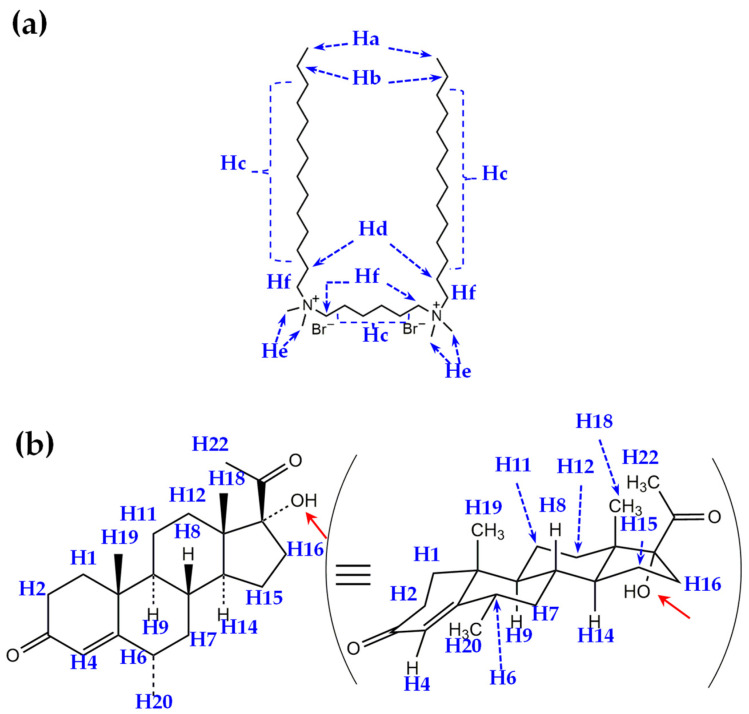
Chemical structures of (**a**) 14-6-14,2Br^−^ and (**b**) MP. The protons of each molecule are labeled as Ha−Hf for the surfactant and H1−H22 for MP. In (**b**), MP is shown with its steroidal backbone, 17α-hydroxy group (indicated by red arrows), and 17β-acetyl group using the chair conformation notations (right) to represent its structure.

Figure 1 illustrates the chemical structures of (a) the gemini surfactant 14-6-14,2Br^−^ and (b) medroxyprogesterone (MP). The protons of each molecule are labeled in the figure to facilitate the discussion of molecular interactions, specifically focusing on the solubilization of MP within the 14-6-14,2Br^−^ micellar system. Protons in 14-6-14,2Br^−^ are denoted as Ha–Hf, whereas those in MP are labeled as H1–H23. The gemini surfactant shown in Figure 1a consists of two long hydrophobic alkyl chains (tetradecyl groups) connected by a short alkyl spacer (hexane) with dimethylammonium bromide headgroups at both ends. This dual structure, characteristic of gemini surfactants, provides a distinct advantage in micellar formation. The hydrophobic alkyl chains facilitate micelle formation in aqueous solutions, resulting in hydrophobic cores that are shielded from the aqueous environment. The hydrophilic headgroups remain exposed to the aqueous solvent, thereby stabilizing the micellar structure. The labeled protons (Ha–Hf) offer crucial insights into the key interactions within the micelle, particularly in the context of solubilizing hydrophobic guest molecules such as MP. In the context of MP solubilization, understanding the specific spatial arrangement of these protons is crucial to elucidate the solubilization mechanism. For example, protons near the headgroups He and Hf are likely to interact with the hydrophilic regions of the solubilized molecules, while protons along the hydrophobic chains Ha, Hb, and Hc can be expected to be in closer proximity to the hydrophobic parts of guest molecules embedded in the micelle core. This differentiation is important when analyzing 2D ROESY spectra, where proton–proton correlations can provide spatial insights into how MP is incorporated into the micellar structure.

Figure 1b shows the chemical structure of MP, a synthetic progestin, which is hydrophobic due to its steroidal backbone. MP is structurally similar to progesterone but features a 17β-acetyl group, which enhances its stability and bioavailability. Notably, the structure of MP can be also expressed using the chair conformation (right), commonly used to represent the three-dimensional structure of steroid molecules. This notation accurately depicts its key functional groups and steroidal framework. The protons of MP, labeled as H1–H22, correspond with different regions of this steroidal framework, including the characteristic methyl groups H8, H18, H19, H20, and the acetyl group H22. These labels allow for the precise identification of MP regions that interact with specific parts of the micelle in NMR and ROESY analyses. For example, the observed cross-peaks in ROESY spectra can reveal which hydrophobic or hydrophilic regions of MP are in close proximity to the micellar components, providing a map of how MP is spatially arranged within the micelle.

The identification of the proton resonances for 14-6-14,2Br^−^ in the ^1^H-NMR spectrum has been thoroughly discussed in previous studies, and the corresponding spectrum can be found in Figure S1 in the Supplementary Materials [14]. For MP, its ^1^H-NMR spectrum in deuterated methanol (CD_3_OD) is presented in Figure S2, and the ^13^C-NMR spectra are shown in Figures S3 and S4. Tetramethylsilane (TMS) at 0 ppm was used as an external reference for ^1^H- and ^13^C-NMR spectra in CD_3_OD. The H1–H22 proton and C1–C22 carbon peaks for MP were assigned using a combination of advanced NMR techniques, including DEPT135 (Figures S5 and S6), ^1^H-^13^C HSQC (Figures S7 and S8), ^1^H-^13^C HMBC (Figures S9 and S10), and ^1^H-^1^H COSY (Figure S11) and NOESY (Figure S12). These assignments provide a comprehensive labeling of MP’s protons (H1–H22), which aids the subsequent discussion on the interaction of MP with the 14-6-14,2Br^−^ micelle.

### 2.2. The 2D ROESY Spectroscopy of MP in Gemini Surfactant Micelles

The critical micelle concentration (cmc) of 14-6-14,2Br^−^ reported in previous studies is approximately 0.16 mM at 298.2 K, with the micellar structure being spherical, a hydrodynamic diameter ranging from 3 to 11 nm, and an aggregation number of around 15 [17]. At concentrations well above the cmc, such as the 2 mM and 5 mM solutions used in this study, micelle formation is fully established, providing an ideal environment for the solubilization of hydrophobic molecules like MP. The cmc plays a crucial role in determining the efficiency of solubilization as micelles only form once the surfactant concentration exceeds the cmc. In our experiments, 2 mM and 5 mM solutions were selected to ensure adequate micelle formation for solubilization and to explore the concentration-dependent effects on solubilization efficiency. Similarly, our previous study with progesterone also indicated that micellar formation at concentrations above the cmc did not significantly alter the micelle’s structural integrity [14]. This consistent behavior across different guest molecules underscores the robustness of 14-6-14,2Br^−^ micelles.

At solutions of 2 mM 14-6-14,2Br^−^, the concentration was approximately 12 times higher than the cmc, indicating that a robust micellar system was present. At 5 mM, the increased concentration of micelles enhanced the system’s solubilization capacity. Notably, the solubilization behavior at these two concentrations showed minimal differences in proton NMR shifts, indicating that the structural integrity of the micelles remained largely unaffected by the surfactant concentration. This observation aligned with previous findings for progesterone [14]. The incorporation of the solute into micelles did not significantly alter the micelle morphology.

**Figure 2:**
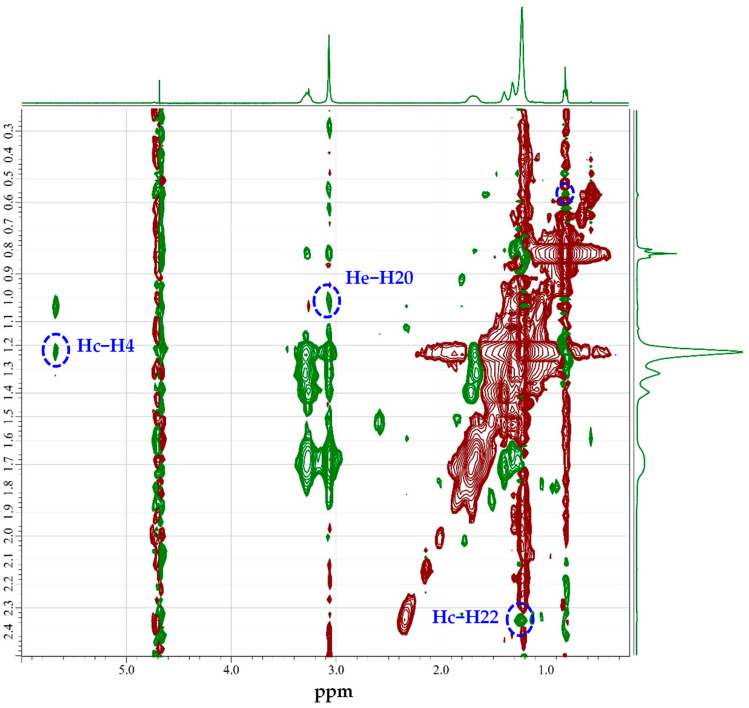
A 2D ROESY spectrum of a 5 mM 14-6-14,2Br^−^ solution with MP maximally solubilized in D_2_O at 298.2 K. Blue dashed regions show correlation signals indicating proximity and interactions between surfactant protons and MP in the micellar system.

Understanding the spatial arrangement of molecules within micelles is crucial to elucidate the solubilization mechanisms of hydrophobic compounds. The use of 2D ROESY spectroscopy provides valuable insights into the proximity and orientation of protons within the micellar system. Figure 2 presents the 2D ROESY spectrum of a 5 mM 14-6-14,2Br^−^ D_2_O solution solubilizing MP at 298.2 K. The blue dashed regions indicate the key correlation signals that revealed the spatial proximity and interactions between the surfactant protons and MP within the micellar system. A key correlation was observed between the protons of the alkyl chain of the surfactant Hc and proton H4 of MP, suggesting a close interaction between the hydrophobic core of the micelle or hydrophilic micellar interface and the steroidal backbone of MP. Another significant correlation was found between the protons near the hydrophilic headgroup of the surfactant He and proton H20 of MP. Additionally, a correlation was observed between the Hc proton of the surfactant and the acetyl group proton H22 of MP.

In contrast, Figure S13 shows the 2D ROESY spectrum of the 2 mM 14-6-14,2Br^−^ solution, offering additional insights into the interactions between MP and the micelles at a lower surfactant concentration. At 2 mM, only two key correlations were observed, between Hc and H4 and between Hc and H22. Although the absence of other correlations seen in the 5 mM solution could suggest a concentration-dependent interaction between MP and the micelles, it is also possible that the reduced intensity of the ROE correlation peaks at a lower surfactant concentration made certain interactions less detectable. The decrease in micelle numbers due to the lower concentration may have led to weaker ROE signals, which might not have been visually apparent despite the interactions still occurring. However, the reduced number of observable interactions at the lower concentration indicated that either fewer regions of MP were engaged in interactions with the micelle or that some correlations were present but too weak to be detected at this concentration.

### 2.3. ROE Correlations between 14-6-14,2Br^−^ and MP at Different Proton Irradiation Positions

**Figure 3:**
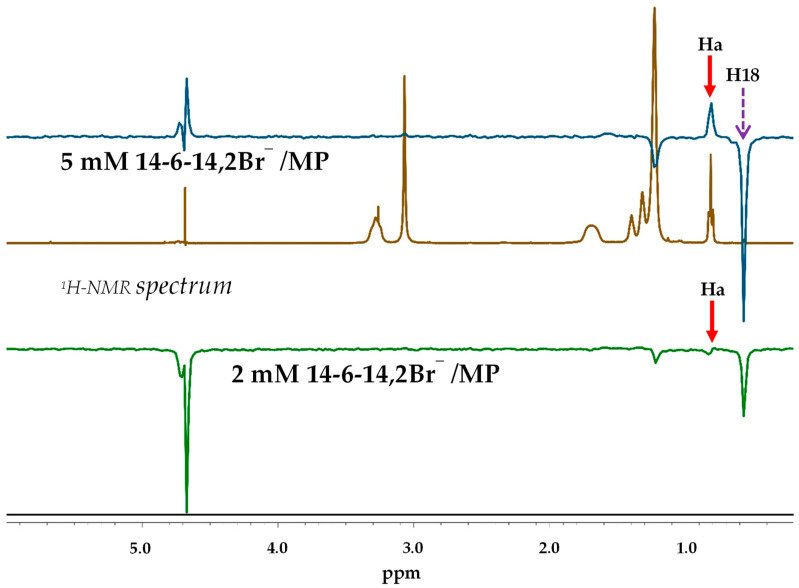
The ROE spectra of 2 mM (**bottom**) and 5 mM 14-6-14,2Br^−^/MP (**top**) of obtained sliced data using one-dimensional processing at diagonal peak H18 and diagonal peaks of 2D ROESY indicating the irradiation positions used to observe ROE correlations. For comparison, the ^1^H-NMR spectrum of 5 mM 14-6-14,2Br^−^/MP is shown in the middle. Purple dotted arrows indicate the proton H18 of MP, and red solid arrows indicate the proton Ha of the surfactant.

Figure 3 presents the one-dimensional ROE spectra derived from the 2D ROESY experiments (Figure 2 and Figure S13), showing the results of irradiation on proton H18 of MP. The spectra correspond with the 2 mM and 5 mM 14-6-14,2Br^−^/MP systems, with the bottom and top spectra representing the 2 mM and 5 mM 14-6-14,2Br^−^ concentrations, respectively. The middle ^1^H-NMR spectrum of the 5 mM system is shown as a comparison spectrum. A positive ROE correlation between proton H18 of MP (indicated by a purple dotted arrow) and proton Ha of the surfactant (indicated by red solid arrows) was observed in both systems, suggesting that MP was localized within or near the hydrophobic core of the micelle. The 5 mM system demonstrated a stronger ROE signal compared with the 2 mM system, indicative of enhanced interaction strength due to the higher concentration of surfactant, which resulted in a greater number of micelles and increased proximity between MP and the surfactant’s hydrophobic chains.

Additional irradiation, detailed in Figures S14–S17, further supported the spatial arrangement of MP within the micelle. When irradiated to proton H4 (Figure S14), a positive ROE peak was observed for Hc in the 2 mM system, while both Hc and He showed ROE peaks in the 5 mM system. This suggests that at lower concentrations, the interaction between H4 and the hydrophobic chain was limited, while higher surfactant concentrations promoted interactions with the spacer regions of the micelle, not with the Hc proton in the hydrophobic chains of the surfactant. Similarly, irradiation to proton H19 (Figure S15) resulted in positive ROE peaks for Hc in the 2 mM system and Hb in the 5 mM system. This indicated that at lower surfactant concentrations, proton H19 primarily interacted with the hydrophobic chain, while at higher concentrations, interactions extended to the terminal methyl groups of the surfactant. The ROE spectra from irradiation to proton H20 (Figure S16) exhibited positive ROE peaks for both Hc and He only in the 5 mM system, suggesting that proton H20 was spatially closer to the spacer regions of the micelle. Lastly, irradiation to proton H22 (Figure S17) resulted in positive ROE peaks for Hc in both the 2 mM and 5 mM systems. The similar behavior across concentrations suggests that proton H22 was consistently positioned near the hydrophobic core of the micelle, which was supported by the ROE correlations between H18 and Ha mentioned above.

The solubilization behavior of MP within 14-6-14,2Br^−^ micelles exhibited distinct differences compared with our previous findings with progesterone. Although both compounds are hydrophobic steroids, MP showed a significantly deeper incorporation into the micellar core, largely due to the presence of the 17β-acetyl groups. In contrast, progesterone primarily localized near the micelle surface. This comparison highlights the importance of structural variations in determining solubilization behavior, which can directly influence the stability and bioavailability of the drug. The enhanced solubilization of MP in gemini surfactants suggests its potential advantages in drug delivery applications, particularly in improving the bioavailability of poorly water-soluble drugs [4,9]. The ROE correlations observed for MP, especially between protons H22 and H19 of MP and proton Hc of the hydrophobic chain of the surfactant, suggest that these regions of MP were positioned deeper within the micelle, closely interacting with the hydrophobic core. This was in contrast to progesterone, where fewer such deep interactions were observed, indicating a more surface-oriented localization [14]. In our previous study, progesterone primarily exhibited ROE correlations with the surfactant headgroups, highlighting its preference for the micelle surface. This limited interaction suggests that progesterone is more likely to reside near the micelle surface, in contrast to MP, which showed deeper incorporation into the micellar core. The stronger correlations between the 17β-acetyl group of H22 of MP with the hydrophobic chain of the surfactant further support the notion that MP embeds more in the micelle core due to the introduction of the 17α-hydroxy group, whereas progesterone primarily interacts with the micelle surface. Additionally, the ROE correlation between the two protons H20 and H4 of MP and proton Hc of the spacer region of the surfactant suggests significant interactions between these parts of MP and the more flexible and hydrophilic parts of the micelle. The presence of these interactions in both the hydrophobic chain and spacer regions highlights the unique positioning of MP, where the 17β-acetyl group of H22 interacted closely with the hydrophobic core while H4 and H20 on its steroidal backbone extended towards the spacer region of the micelle. This contrasted with progesterone, which primarily localized near the surface of the micelle, interacting more extensively with the micelle headgroups and showing fewer interactions with the hydrophobic core.

### 2.4. Machine Learning and Computational Analyses

**Figure 4:**
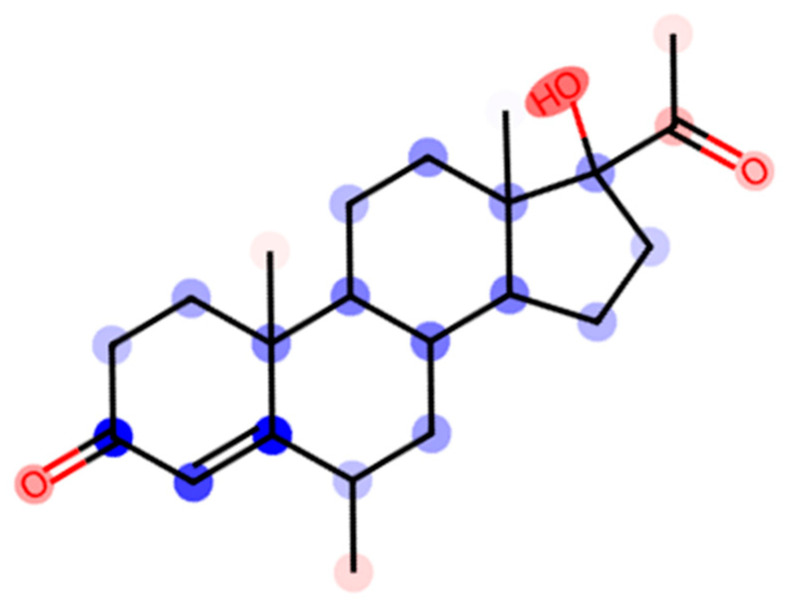
Structural fingerprint of MP highlighting the contribution of different structural elements to its solubility (log*S*) in water. The elements contributing positively to the solubility are highlighted in red, while those contributing negatively are in blue. The intensity of the color corresponds with the magnitude of the contribution. This fingerprint provides a visual representation of how different parts of the progesterone molecule influence its solubility behavior.

Figure 4, generated using machine learning models to predict solubility contributions, offers a structural fingerprint of MP. In this fingerprint, elements contributing positively to solubility are highlighted in red, while those contributing negatively are in blue. The intensity of the color corresponds with the magnitude of the contribution [14]. Notably, proton H20, situated near carbon C6 where a methyl group was attached, is highlighted in red, indicating its hydrophilic nature. This contrasts with many other protons in the steroid backbone, which tend to exhibit more hydrophobic characteristics. The methyl group attached to carbon C6 is a distinguishing feature of MP when compared with progesterone, which lacks this substituent. The hydrophilic nature of proton H20, as suggested by its red coloration in Figure 4, was consistent with the ROE correlations observed in the 2D ROESY spectra. Specifically, the interactions between proton H20 of MP and the spacer and headgroup protons Hc and He of the 14-6-14,2Br^−^ micelles indicated that proton H20 was located near the micelle surface. Furthermore, the correlations between proton H4 and the spacer proton Hc, as well as between proton H4 and the headgroup proton He, further supported this spatial arrangement. These correlations suggest that both protons H20 and H4 were oriented towards the micelle surface, where they interacted with the more hydrophilic regions of the surfactant.

Another important structural feature of MP is the 17α-hydroxy group. This group, known for its strong hydrophilic properties, would be expected to further enhance the solubility of MP by interacting with the hydrophilic micelle surface. Yet, based on the chair conformation of MP as shown in Figure 1, the hydroxy group at C17 was oriented in the opposite direction to the acetyl group at the C17β position. This spatial arrangement minimized the potential for the hydroxy group to contribute to the hydrophilicity near the acetyl group as its positioning reduced its exposure to the micelle surface. Consequently, the expected enhancement of hydrophilicity around the acetyl group due to the introduction of the hydroxy group was not observed as strongly as was anticipated. In contrast, the majority of the steroidal backbone of MP, including proton H22 of the 17β-acetyl group, interacted more closely with the hydrophobic core of the micelle, as evidenced by the strong ROE correlations with proton Hc on the hydrophobic chain of the surfactant. This dual localization—where the steroid backbone resided within the hydrophobic core and the methyl group linked to the C6 position extended towards the micelle surface—highlighted the distinct solubilization behavior of MP compared with progesterone.

In the case of progesterone, our previous studies demonstrated that it primarily localizes near the micelle surface, extensively interacting with the surfactant headgroups through its acetyl group. However, the absence of the methyl group at the C6 position in progesterone resulted in fewer correlations with the spacer and hydrophobic regions of the micelle. This difference emphasizes the significant role of the methyl group at the C6 position in MP, which promotes more extensive interactions with both the spacer and headgroup regions of the surfactant. The enhanced solubilization behavior of MP, particularly at higher surfactant concentrations, can be attributed to this unique structural feature as it allows for a more stable incorporation of MP within the micellar structure.

The electron density map presented in Figure S18 highlights a notable difference between the electron distributions of MP and progesterone, particularly in the acetyl group. As seen in the figure, the acetyl oxygen in progesterone exhibits high electron density, indicated by a red arrow, suggesting a strong ability to engage in hydrophilic interactions. In contrast, the acetyl oxygen in MP shows significantly lower electron density, shown by a red arrow, reflecting its reduced capacity for hydrophilic interactions due to the introduction of the hydroxy group at the C17α position. This introduction of the hydroxy group in MP alters the electron distribution across the molecule. As a result, the overall electron density at the C3 position in MP is relatively higher than in progesterone, which affects the molecule’s hydrophilic behavior. The increased electron density at this site enhances the interaction of MP with the hydrophilic regions of the micelle, particularly near the micelle surface, further supporting the observed solubilization behavior.

These findings corroborated the spatial arrangement proposed in the ROESY data, where the methyl group at the C6 position and the hydrophilic regions of MP, such as the carbonyl group at the C3 position, interacted more significantly with the micelle surface compared with progesterone. The introduction of the hydroxy group in MP, while not directly contributing to hydrophilicity near the acetyl group, nonetheless shifted the solubilization dynamics, promoting stronger interactions with the micelle’s hydrophilic regions. This behavior contrasted with that of progesterone, which lacks the methyl group at the C6 position and shows fewer interactions with the surfactant’s spacer regions. Thus, the unique solubilization properties of MP within gemini surfactant micelles can be attributed to this distinct electron density distribution. This difference enhances MP’s interaction with the micelle surface, supporting its incorporation into the micellar structure and offering insights into its enhanced solubilization compared with progesterone.

## 3. Materials and Methods

### 3.1. Materials

The gemini surfactant, hexanediyl-1-6-bis(dimethyltetradecylammonium bromide (14-6-14,2Br^−^), was synthesized and purified in our laboratory following the procedure described in previous works [17,25]. Medroxyprogesterone (MP; purity > 98%) was obtained from Tokyo Chemical Industry (Tokyo, Japan). All chemicals were used as received without further purification. Deuterium oxide (D_2_O) and deuterated methanol (CD_3_OD) for the NMR studies were purchased from Merck KGaA (Darmstadt, Germany).

### 3.2. Preparation of Surfactant Solutions and MP Solubilization

14-6-14,2Br^−^ was dissolved in D_2_O to prepare solutions at 2.0 and 5.0 mmol dm^−3^ (or mM). For NMR measurements, MP was added to the surfactant solution until it reached its saturation concentration. The mixtures were then kept constant at 298.2 K and stirred to ensure the complete solubilization of MP and to reach a solubilization equilibrium. After reaching an equilibrium, the solutions were filtered through a membrane filter of 0.1 μm pore size (Millipore MILLEX VV, Merck KGaA) to remove extra undissolved materials. This procedure was performed to maximize the solubilization of MP in the surfactant solutions, where the maximum solubilization concentration of MP was found to be 0.23 mM in a 2 mM 14-6-14,2Br^−^ solution and 0.51 mM in a 5 mM 14-6-14,2Br^−^ solution [26].

### 3.3. NMR Spectroscopy

NMR spectra were recorded using a JEOL JNM-ECZ400S (JEOL Ltd., Tokyo, Japan) operating at 400 MHz. The chemical shifts were reported in parts per million (ppm) and the chemical shifts of acetonitrile used as an external reference were 1.98 ppm for ^1^H- and 1.7 ppm for ^13^C-NMR spectra. The 2D ROESY experiments were performed using a mixing time of 250 ms and a relaxation delay of 1.884 s. The temperature was maintained at 298.2 K during all NMR spectra measurements [14].

### 3.4. Structural Fingerprint Generation and Analysis

The structural fingerprint of MP was generated and analyzed using Python libraries and packages, including PyCaret (ver. 3.0.4) and RDKit (ver. 2023.3.2). The dataset used in this study was derived from a logarithm of water solubility (log*S*) dataset of 1290 compounds published by T.J. Hou et al. [27]. The Extended Connectivity Fingerprint (ECFP4) was computed for each molecule in the list, and the resulting fingerprints were converted into integer-type NumPy arrays and stored in a Pandas DataFrame. The ‘log*S*’ property of each molecule, representing its solubility, was also added to DataFrame. A Bayesian ridge model, which provides the coefficients of individual features (the magnitude of influence each characteristic has on the target variable), was created and tuned to optimize the root mean square error (RMSE), and a final model was created using all the data. The final model achieved an RMSE of 1.18 and an *R*^2^ of 0.66. The Simplified Molecular Input Line Entry System (SMILES) notation for progesterone was converted into a molecular object and the ECFP4 fingerprint was computed. The fingerprint was passed to the regression model to predict the solubility of MP. The predicted value was found to be log*S* = −5.03 (−4.73 for the experimental value at 298.2 K in the previous study [26]). The contributions of the bits to the fingerprint were calculated, and the atoms contributing to the fingerprint were highlighted [28]. The atoms with positive contributions were colored in shades of red and those with negative contributions were colored in shades of blue.

### 3.5. Electron Density Map Generation

The electron density map of MP was generated using the Psi4 package (ver. 1.7) in Python, an open-source suite of ab initio quantum chemistry programs. The SMILES notation for MP was converted into a XYZ format, which is a common format used to represent atom coordinates in 3D. The molecule was then optimized using the UFF (Universal Force Field) method. The Hartree–Fock method with an STO-3G basis set was used to compute the energy of the molecule. The resulting wavefunction was stored and the variables in the wavefunction were printed for reference. The Mulliken charges were calculated for a basis set of STO-3G. The Similarity Maps function from the rdkit (version: 2024.03.5). Chem.Draw module was used to generate similarity maps for each basis set. The maps were colored using the ‘RdBu’ colormap (matplotlib: version 3.7.1).

## 4. Conclusions

This study examined the solubilization behavior of medroxyprogesterone (MP) within micelles formed by the gemini surfactant 14-6-14,2Br^−^ in comparison with previously studied progesterone. NMR spectroscopy revealed that MP exhibits a distinct solubilization pattern, embedding more deeply into the micellar core than progesterone, which primarily localizes near the micelle surface. This difference was attributed to the unique structural features of MP, particularly the 6α-methyl group and the hydroxy group at the C17 position, which enhanced its interactions with the hydrophobic regions of the micelles. ROE correlations, especially between two protons of MP (H22 and H19) and the hydrophobic chains of the surfactant, further emphasized this deeper incorporation within the micellar structure. Machine learning and electron density analyses supported these findings, highlighting the significant role of the structural elements of MP, such as the methyl group at the C6 position, in its solubilization dynamics. The enhanced solubilization capacity of MP was thus linked to these specific structural characteristics, contrasting with the more surface-oriented localization of progesterone. These insights deepen our understanding of the interaction mechanisms between hydrophobic compounds and gemini surfactant micelles. Moreover, the comparison of MP with progesterone underscores the significance of subtle structural modifications in influencing the solubilization dynamics within micelles. This knowledge is crucial for the rational design of drug delivery systems that aim to optimize the solubilization, stability, and bioavailability of hydrophobic pharmaceutical compounds. Furthermore, they underscore the potential of 14-6-14,2Br^−^ micelles in accommodating structurally diverse hydrophobic molecules, thereby advancing drug delivery applications.

## Data Availability

Data will be made available on request.

## Supplementary Materials

The following supporting information can be downloaded at https://www.mdpi.com/article/10.3390/molecules29204945/s1. Figure S1: ^1^H-NMR spectrum of 14-6-14,2Br^−^ in D_2_O at 298.2 K; Figure S2: ^1^H-NMR spectrum of MP in CD_3_OD at 298.2 K; Figure S3: ^13^C-NMR spectrum of MP in CD_3_OD at 298.2 K; Figure S4: ^13^C-NMR spectrum of MP in CD_3_OD at 298.2 K ranging from 14.8 to 58.6 ppm; Figure S5: DEPT135 (top) and ^13^C-NMR (bottom) spectra of MP in CD_3_OD at 298.2 K highlighting the differentiation between the CH, CH_2_, and CH_3_ carbon types; Figure S6: DEPT135 (top) and ^13^C-NMR (bottom) spectra of MP in CD_3_OD at 298.2 K highlighting the differentiation between the CH, CH_2_, and CH_3_ carbon types; Figure S7: Correlation signals between ^1^H (0.1 to 6.2 ppm) and ^13^C (5 to 132 ppm) in ^1^H-^13^C HSQC spectrum of MP in CD_3_OD at 298.2 K; Figure S8: Correlation signals between ^1^H (0.4 to 2.72 ppm) and ^13^C (11 to 61 ppm) in ^1^H-^13^C HSQC spectrum of MP in CD_3_OD at 298.2 K; Figure S9: Correlation signals between ^1^H (0.78 to 2.37 ppm) and ^13^C (167 to 226 ppm) in ^1^H-^13^C HMBC spectrum of MP in CD_3_OD at 298.2 K; Figure S10: Correlation signals between ^1^H (0.46 to 1.41 ppm) and ^13^C (28 to 60 ppm) in ^1^H-^13^C HMBC spectrum of MP in CD_3_OD at 298.2 K; Figure S11: Correlation signals between ^1^H (0.2 to 2.9 ppm) and ^1^H (0.2 to 2.9 ppm) in ^1^H-^1^H COSY spectrum of MP in CD_3_OD at 298.2 K; Figure S12: Correlation signals between ^1^H (0.4 to 2.9 ppm) and ^1^H (0.4 to 2.9 ppm) in NOESY spectrum of MP in CD_3_OD at 298.2 K; Figure S13: ROESY spectrum of 2 mM 14-6-14,2Br^−^ solution with MP maximally solubilized in D_2_O at 298.2 K; Figure S14: The ROE spectra of 2 mM (bottom) and 5 mM 14-6-14,2Br^−^/MP (top) of obtained sliced data from one-dimensional processing at diagonal peak H4; Figure S15: The ROE spectra of 2 mM (bottom) and 5 mM 14-6-14,2,Br^−^/MP (top) of obtained sliced data from one-dimensional processing at diagonal peak H19; Figure S16: The ROE spectra of 2 mM (bottom) and 5 mM 14-6-14,2Br^−^/MP (top) of obtained sliced data from one-dimensional processing at diagonal peak H20; Figure S17: The ROE spectra of 2 mM (bottom) and 5 mM 14-6-14,2Br^−^/MP (top) of obtained sliced data from one-dimensional processing at diagonal peak H22; Figure S18: Electron density map of MP (left) and progesterone (right) calculated using Psi4 with the Mulliken charge scheme and the STO-3G basis set.
