# Supplementary material for: Distinct Solubilization Mechanisms of Medroxyprogesterone in Gemini Surfactant Micelles: A Comparative Study with Progesterone
Source: Molecules. 2024 Oct 19;29(20):4945. doi: 10.3390/molecules29204945 (PMC11510562; doi:10.3390/molecules29204945)
Supplement: Supplementary file 1 [file molecules-29-04945-s001.zip › molecules-3252343-supplementary.pdf]

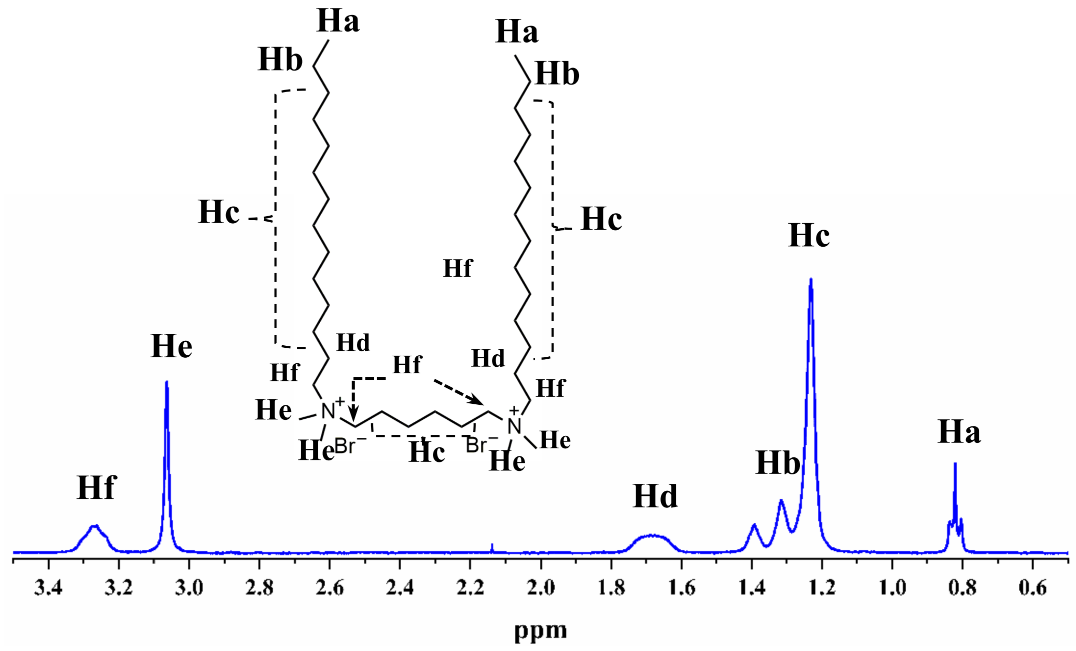


**Fig. S1** $^{1}H$-NMR spectrum of 14-6-14, $2Br^{-}$ in $D_2O$ at 298.2K.

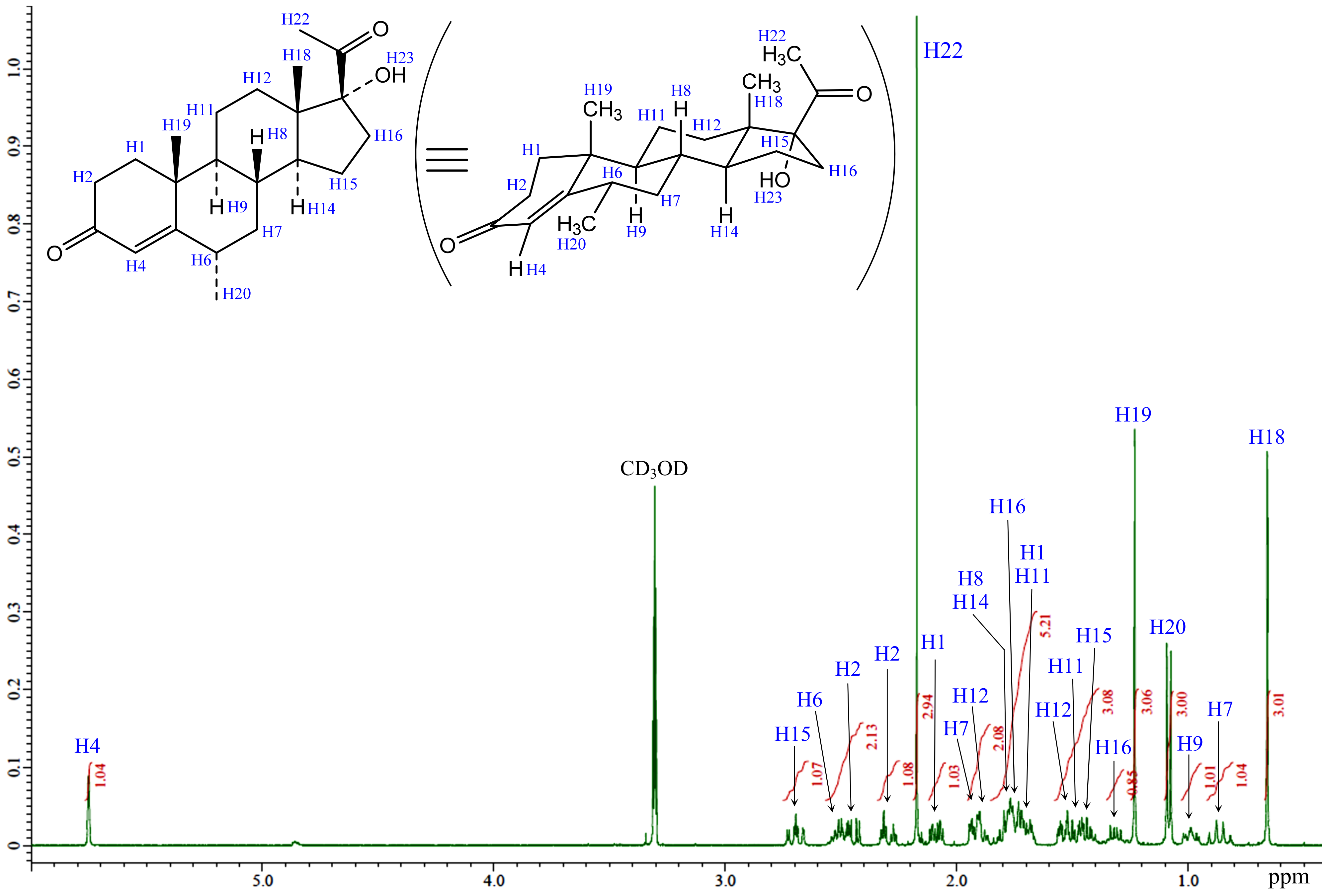


**Fig. S2** $^{1}$H-NMR spectrum of MP in $CD_3OD$ at 298.2K.

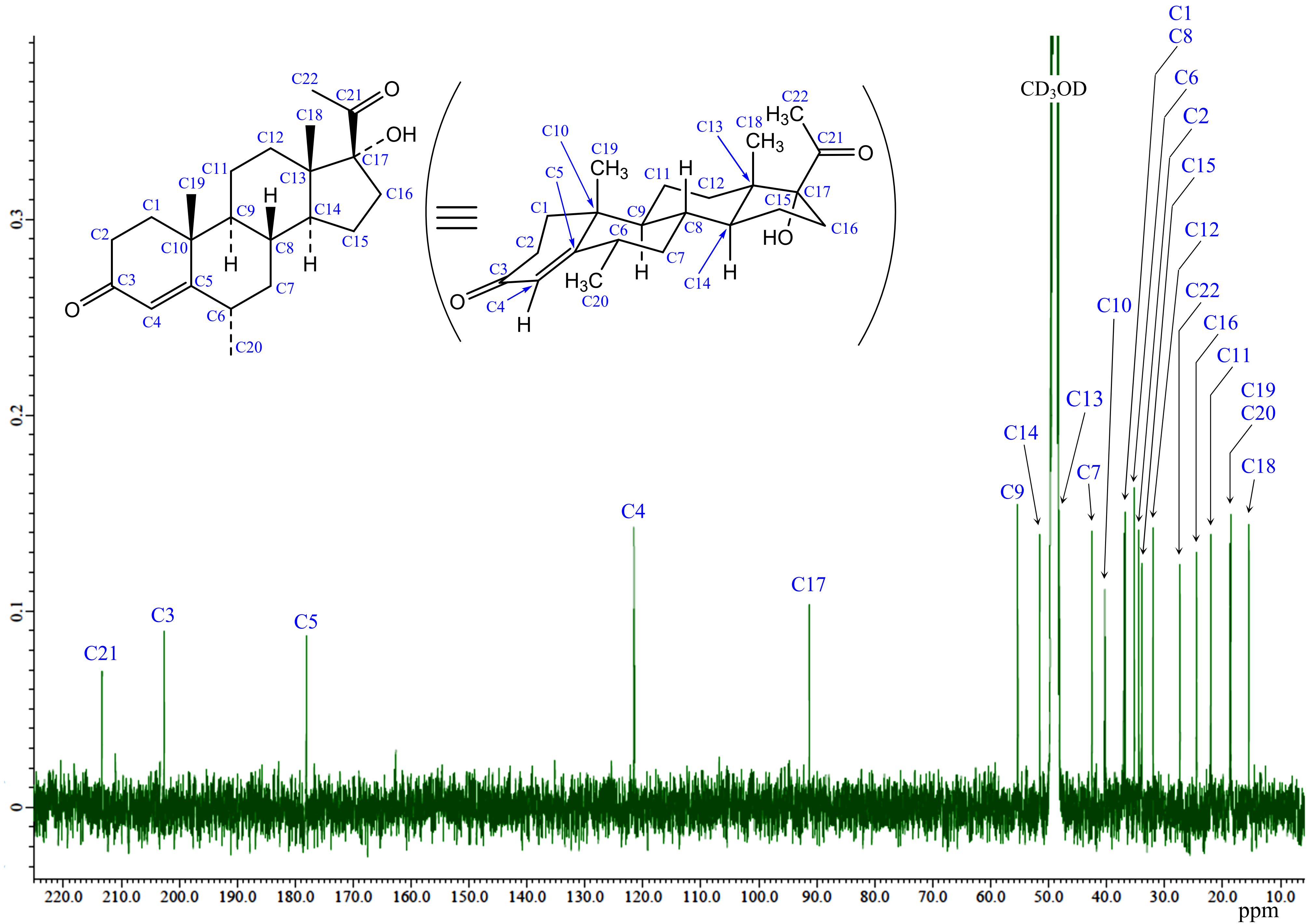


**Fig. S3** $^{13}$C-NMR spectrum of MP in $CD_3OD$ at 298.2K.

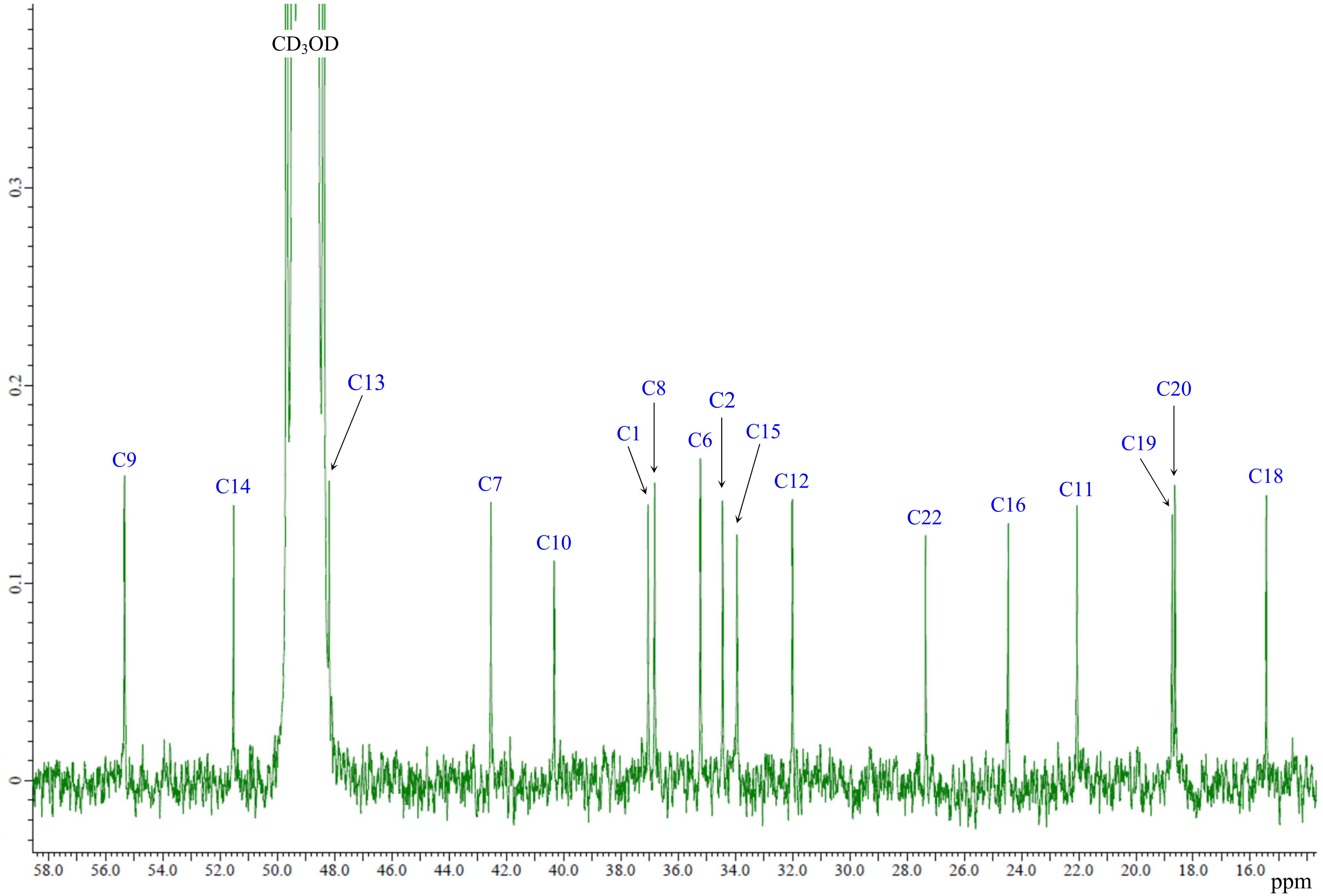


**Fig. S4** $^{13}$C-NMR spectrum of MP in $CD_3OD$ at 298.2K, ranging from 14.8 to 58.6 ppm.

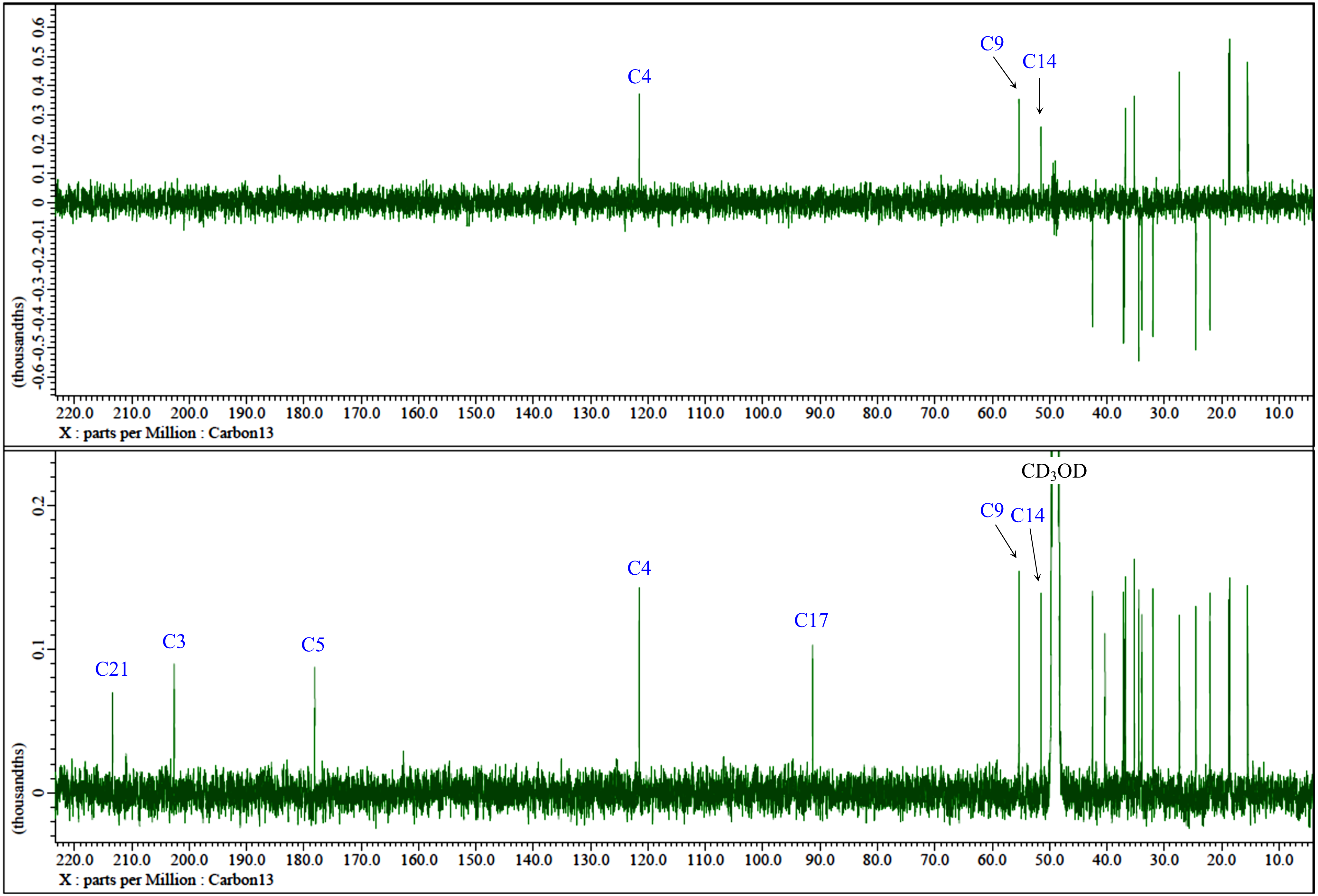


**Fig. S5** DEPT135 (top) and $^{13}$C-NMR (bottom) spectra of MP in $CD_3OD$ at 298.2K, ranging from 4 to 224 ppm, highlighting the differentiation of CH, $CH_2$, and $CH_3$ carbon types.

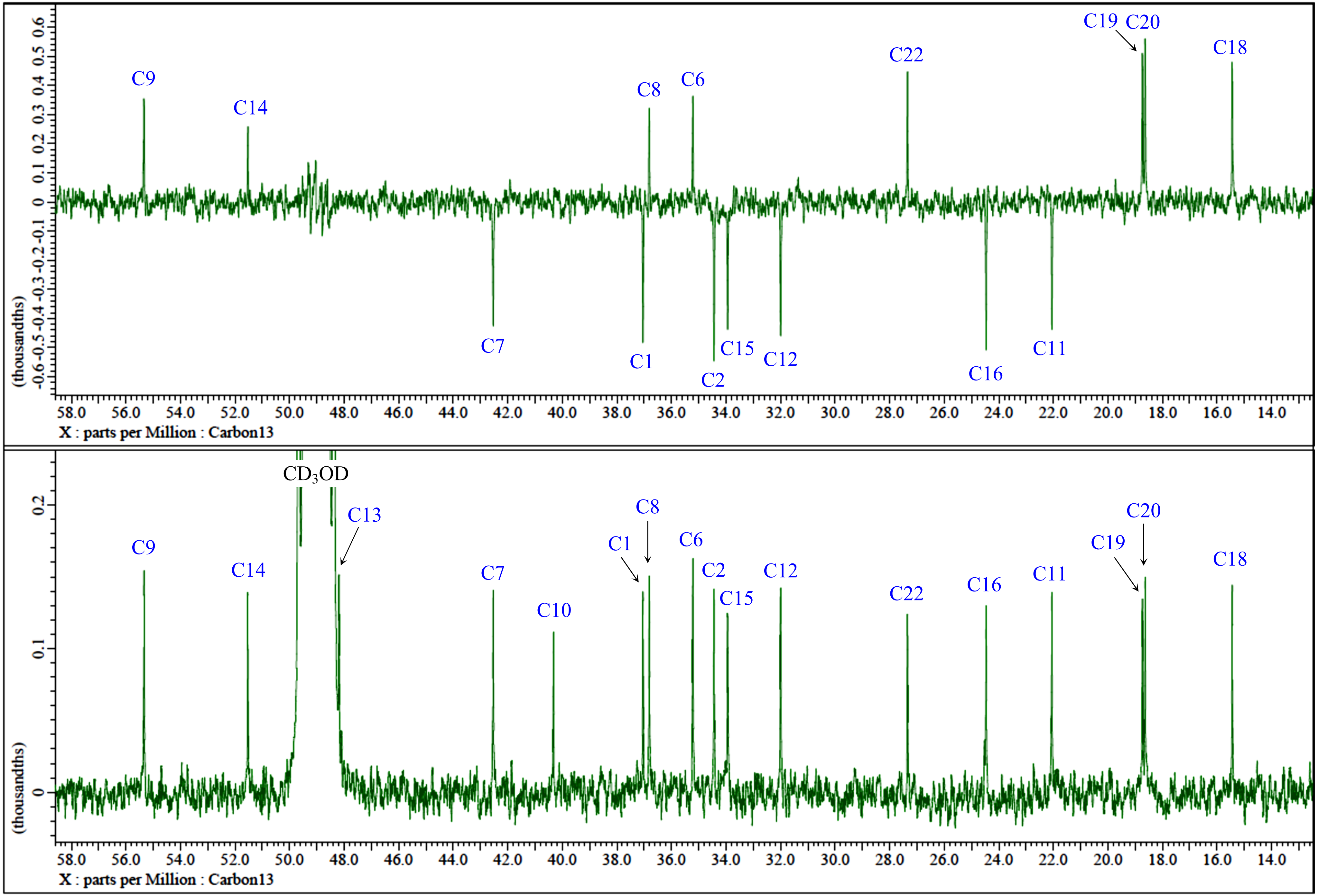


**Fig. S6** DEPT135 (top) and $^{13}$C-NMR (bottom) spectra of MP in $CD_3OD$ at 298.2K, ranging from 12.6 to 58.6 ppm, highlighting the differentiation of CH, $CH_2$, and $CH_3$ carbon types.

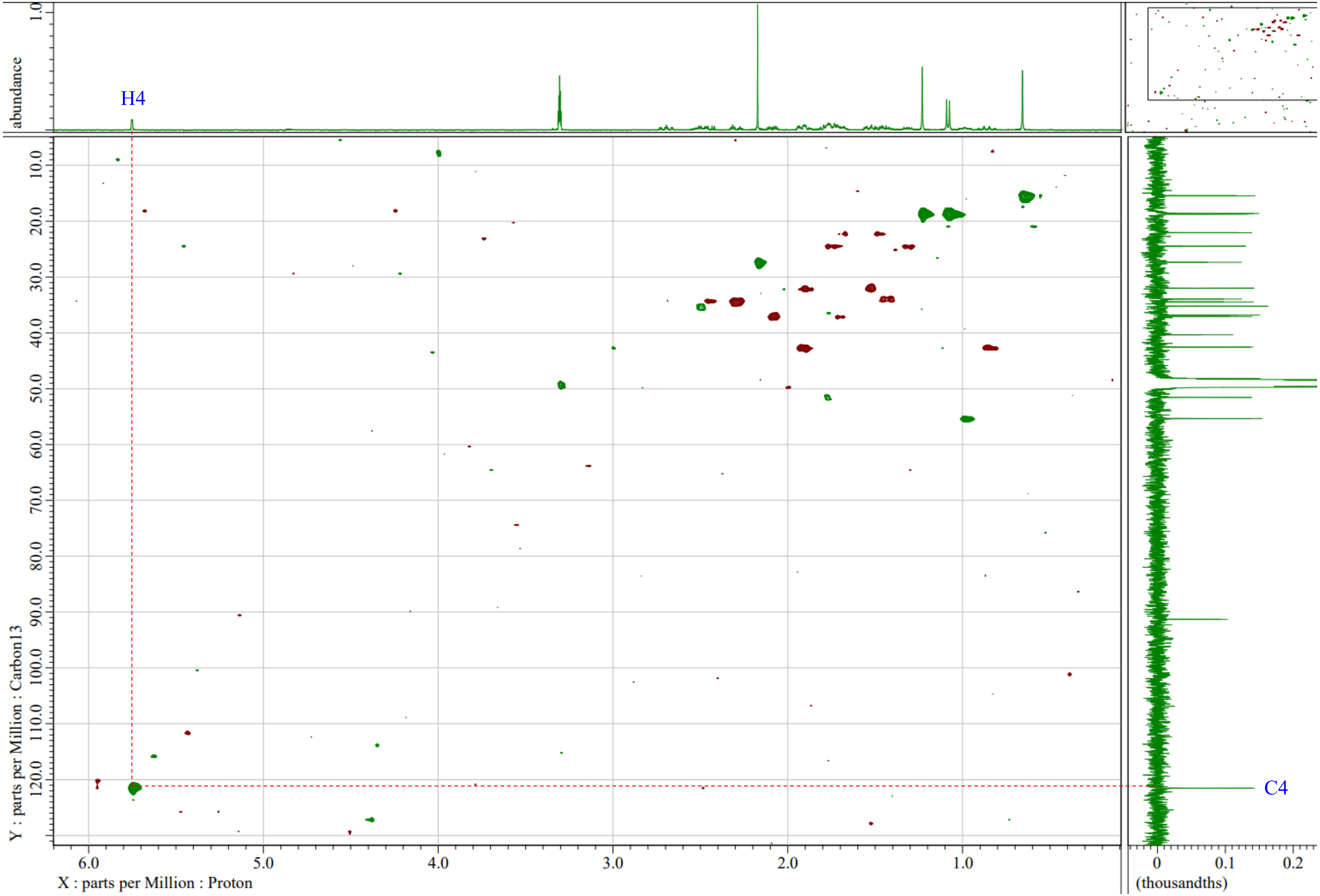


**Fig. S7** Correlation signals between $^{1}$H (0.1 to 6.2 ppm ) and $^{13}$C (5 to 132 ppm) in $^{1}$H-$^{13}$C HSQC spectrum of MP in $CD_3OD$ at 298.2K.

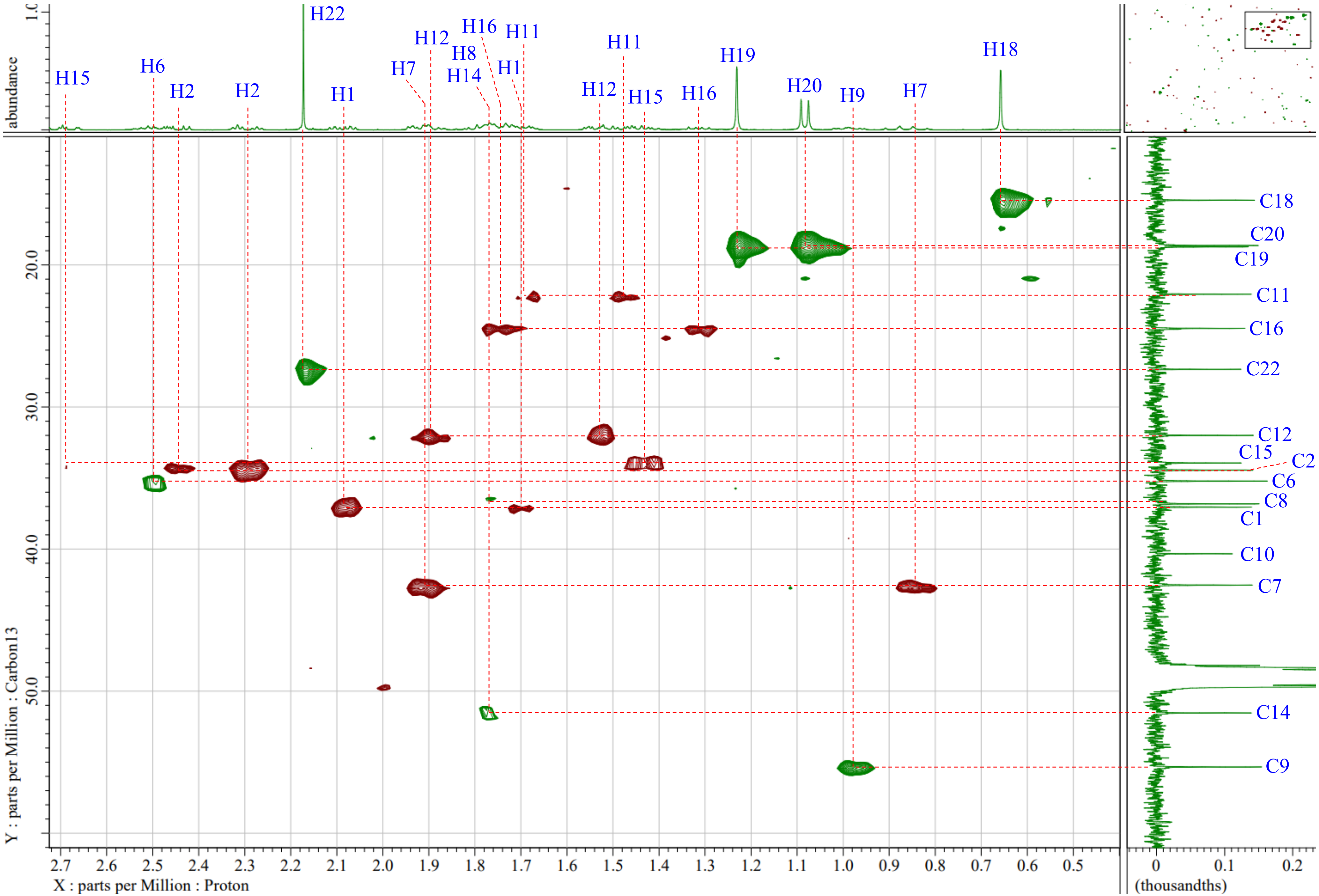


**Fig. S8** Correlation signals between $^{1}$H (0.4 to 2.72 ppm ) and $^{13}$C (11 to 61 ppm) in $^{1}$H-$^{13}$C HSQC spectrum of MP in $CD_3OD$ at 298.2K.

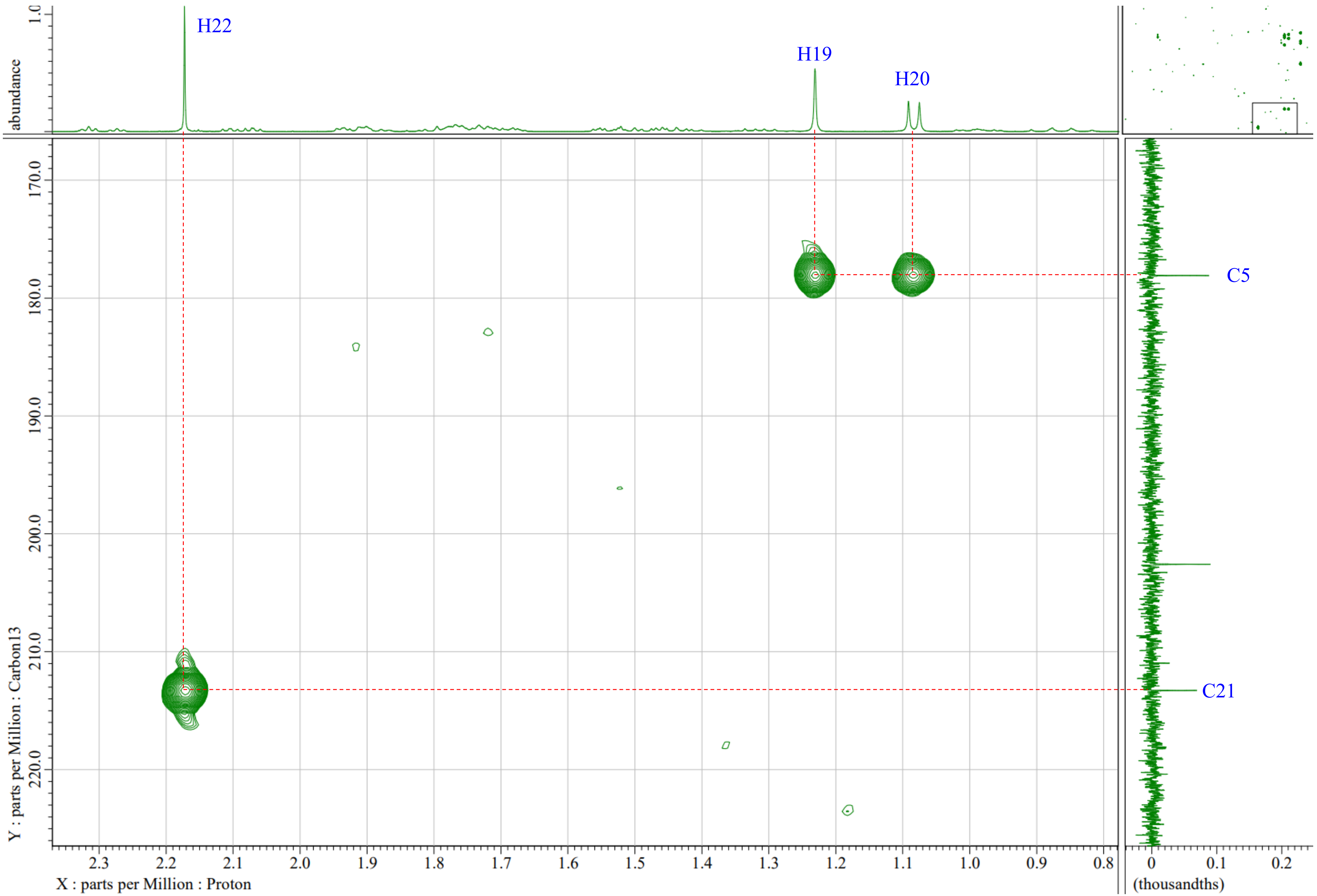


**Fig. S9** Correlation signals between $^{1}H$ (0.78 to 2.37 ppm ) and $^{13}C$ (167 to 226 ppm) in $^{1}H$-$^{13}C$ HMBC spectrum of MP in $CD_3OD$ at 298.2K.

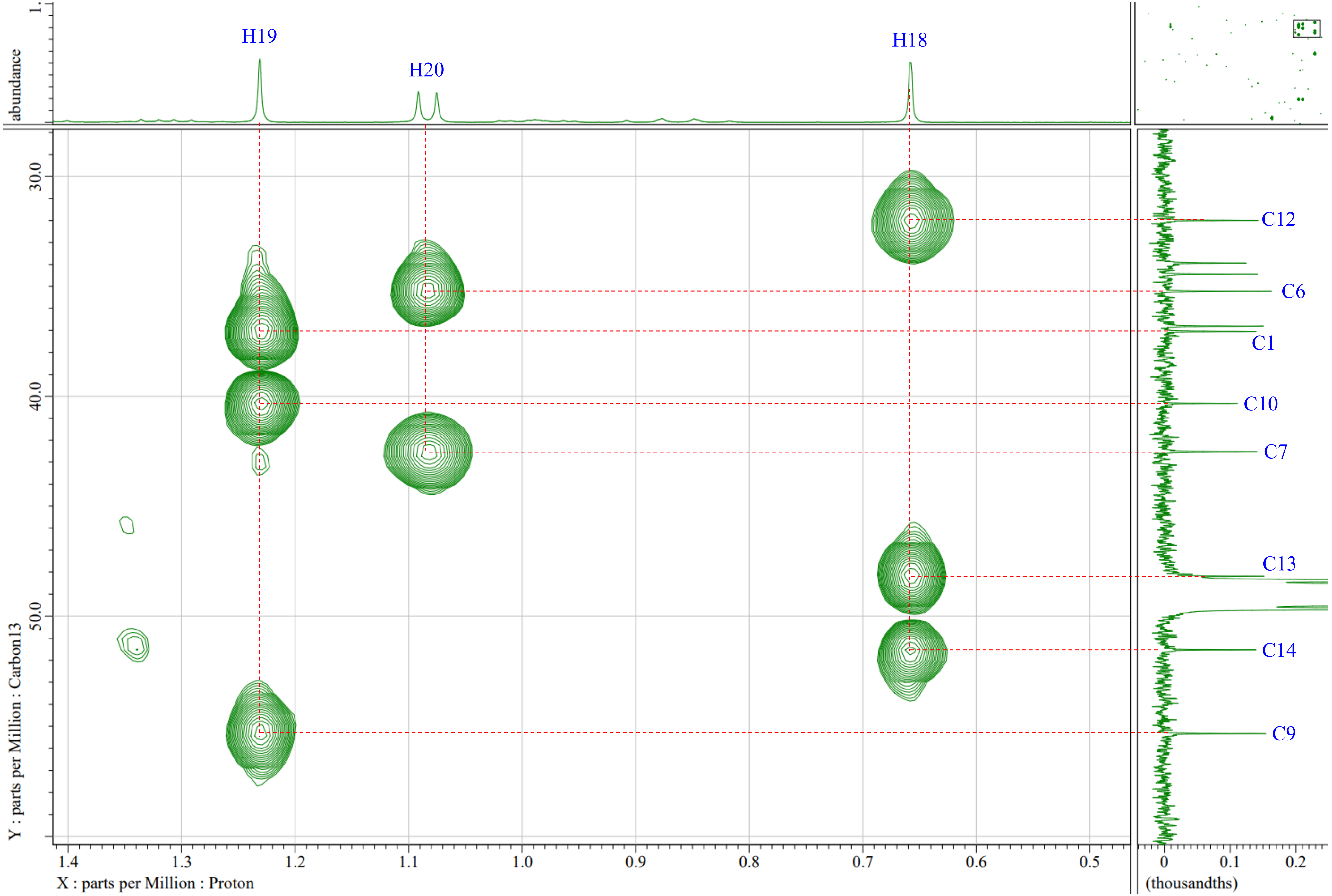


**Fig. S10** Correlation signals between $^{1}H$ (0.46 to 1.41 ppm) and $^{13}C$ (28 to 60 ppm) in $^{1}H$-$^{13}C$ HMBC spectrum of MP in $CD_3OD$ at 298.2K.

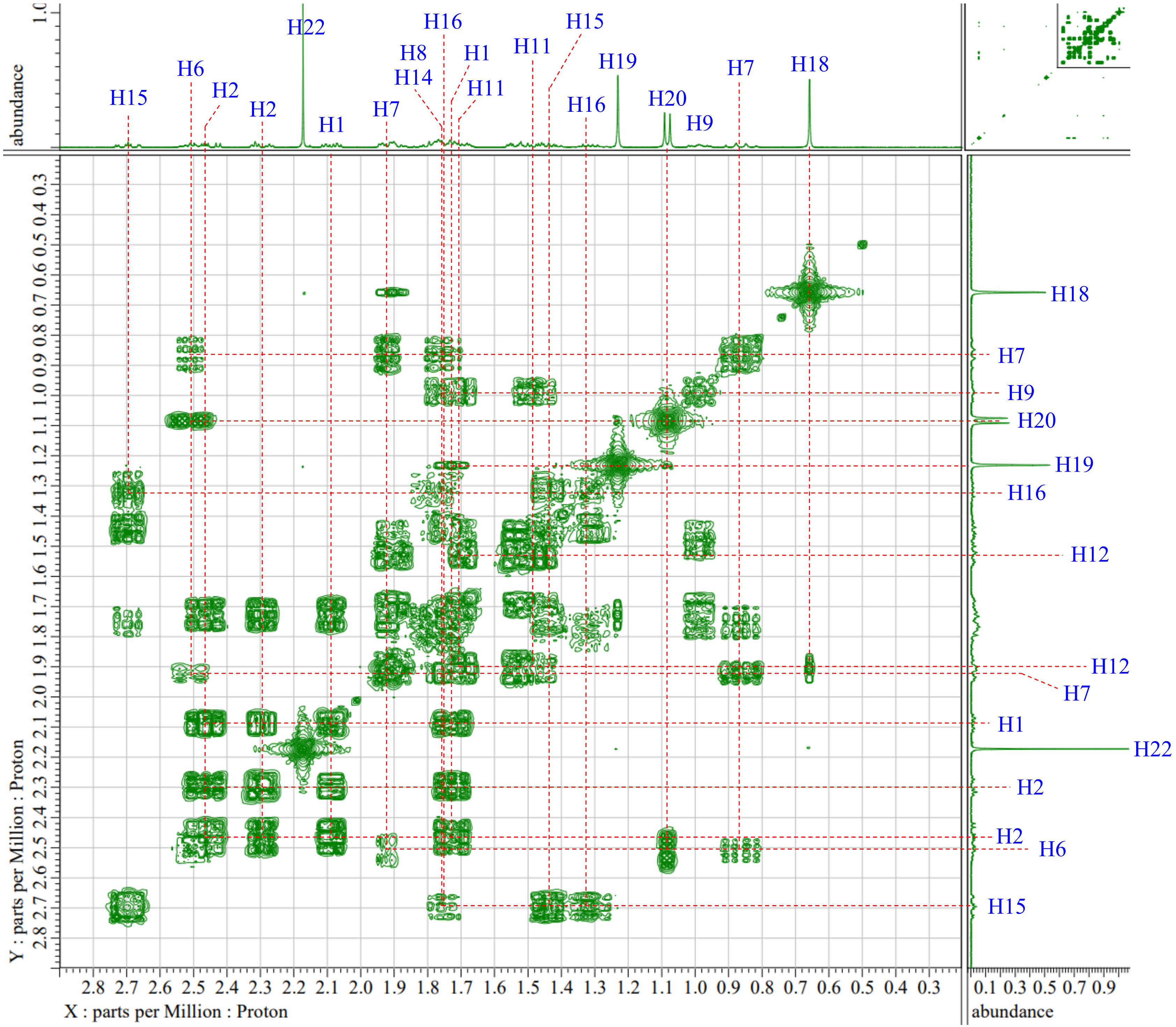


**Fig. S11** Correlation signals between $^{1}H$ (0.2 to 2.9 ppm ) and $^{1}H$ (0.2 to 2.9 ppm) in $^{1}H$-$^{1}H$ COSY spectrum of MP in $CD_3OD$ at 298.2K.

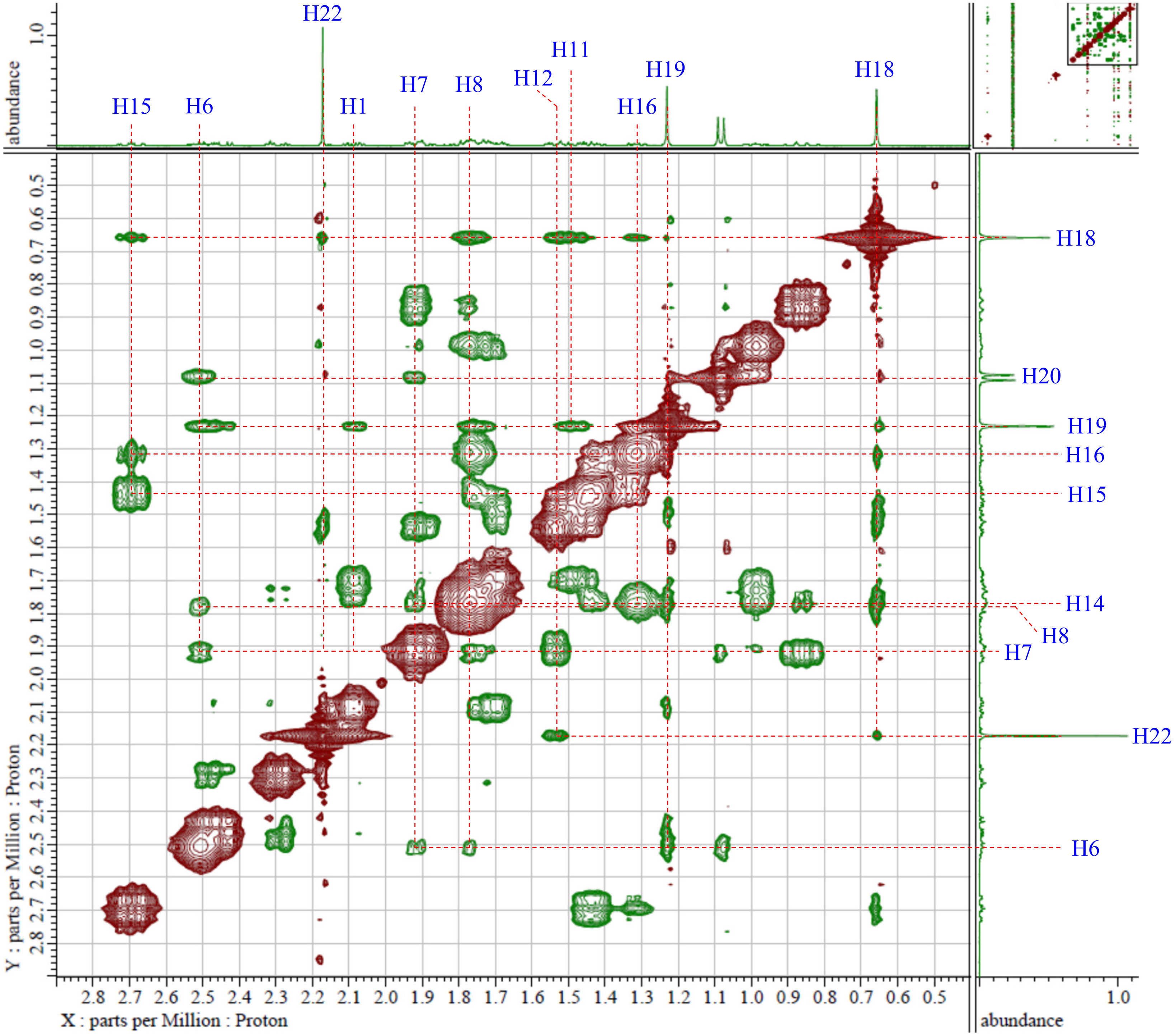


**Fig. S12** Correlation signals between $^{1}H$ (0.4 to 2.9 ppm ) and $^{1}H$ (0.4 to 2.9 ppm) in NOESY spectrum of MP in $CD_3OD$ at 298.2K.

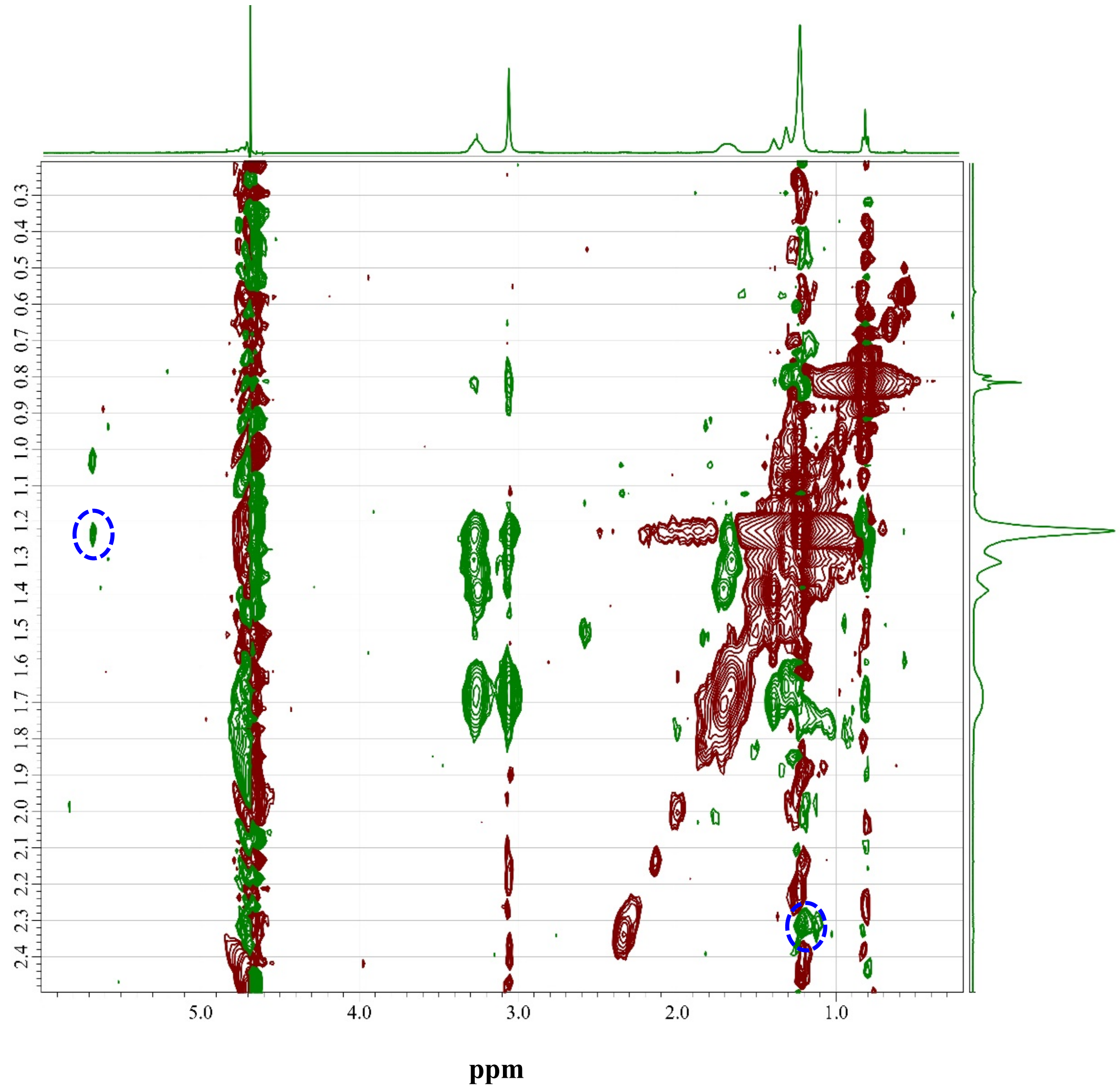


**Fig. S13** ROESY spectrum of 2 mM 14-6-14,2Br$^-$ solution with MP maximally solubilized in $D_2O$ at 298.2 K.

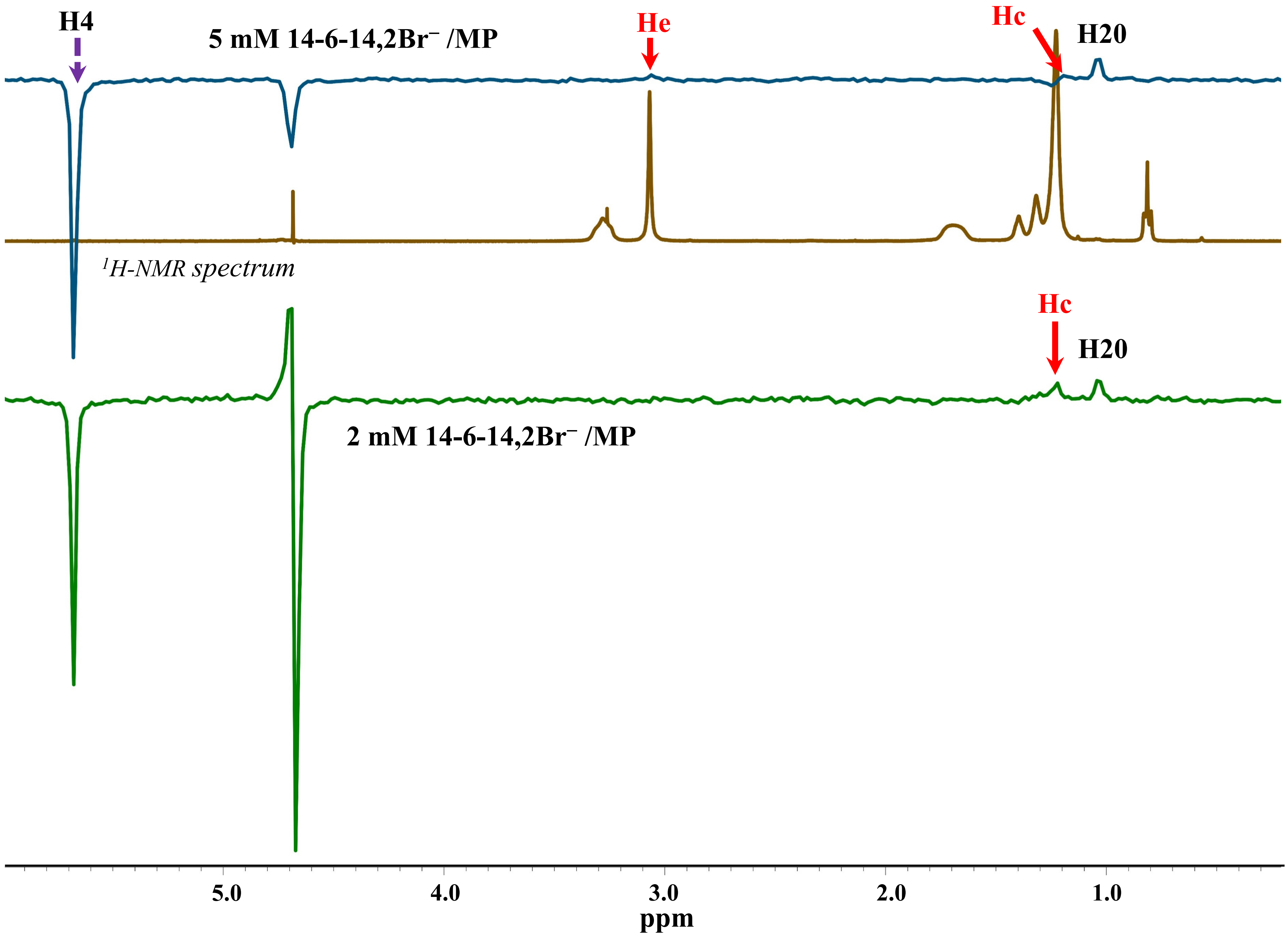


**Fig. S14** The ROE spectra of 2 mM (bottom) and 5 mM 14-6-14,$2Br^-$/MP (top) of obtained sliced data by one-dimensional processed at diagonal peak H4, diagonal peaks of 2D ROESY that mean irradiation positions for observing ROE correlations. For comparison, the $^1$H-NMR spectrum of 5 mM 14-6-14,$2Br^-$/MP is shown in the middle.

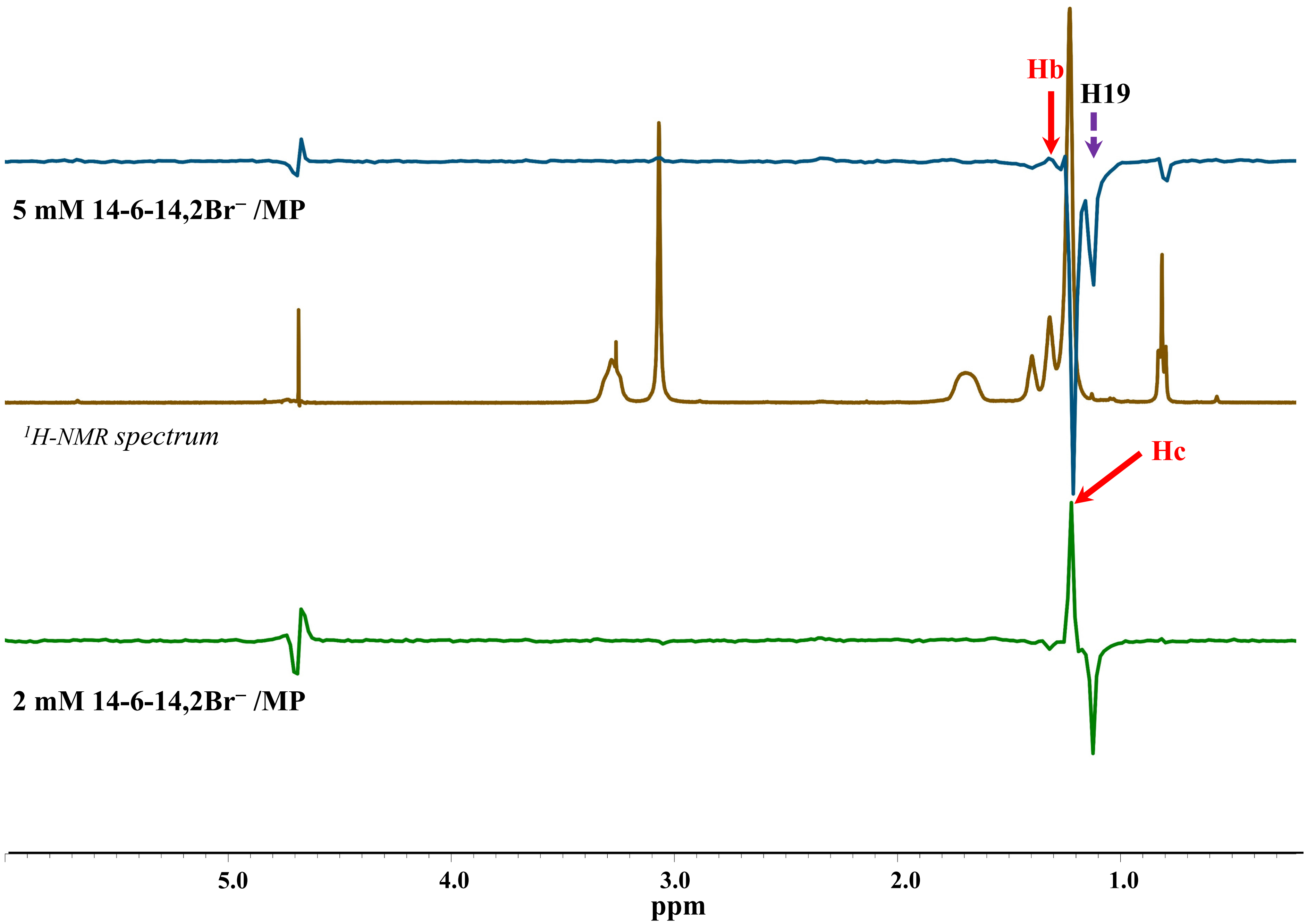


**Fig. S15** The ROE spectra of 2 mM (bottom) and 5 mM 14-6-14,$2Br^-$/MP (top) of obtained sliced data by one-dimensional processed at diagonal peak H19, diagonal peaks of 2D ROESY that mean irradiation positions for observing ROE correlations. For comparison, the $^1$H-NMR spectrum of 5 mM 14-6-14,$2Br^-$/MP is shown in the middle.

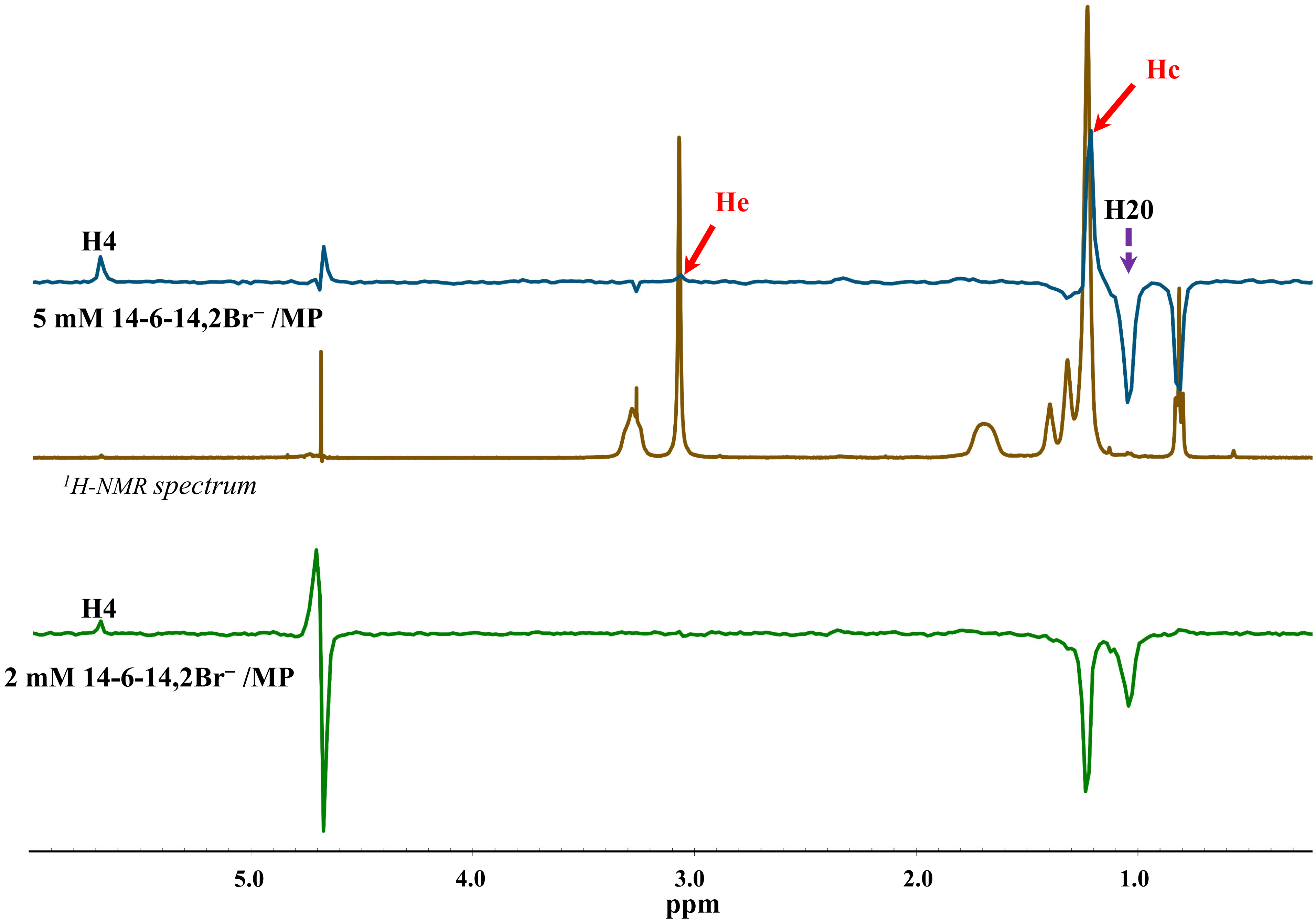


**Fig. S16** The ROE spectra of 2 (bottom) and 5 mM 14-6-14,2Br$^{-}$/MP (top) of obtained sliced data by one-dimensional processed at diagonal peak H20, diagonal peaks of 2D ROESY that mean irradiation positions for observing ROE correlations. For comparison, the $^{1}$H-NMR spectrum of 5 mM 14-6-14,2Br$^{-}$/MP is shown in the middle.

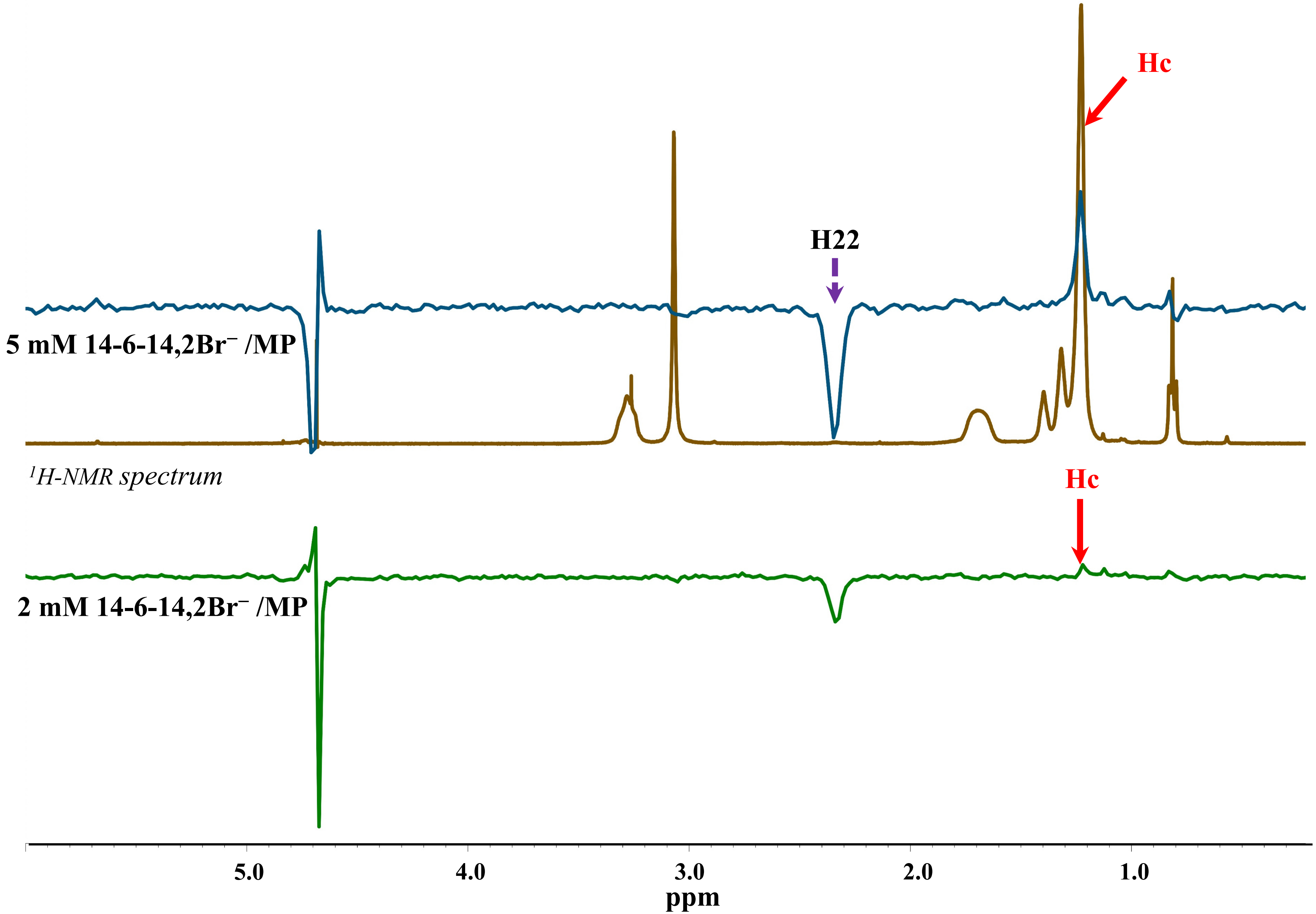


**Fig. S17** The ROE spectra of 2 (bottom) and 5 mM 14-6-14,2$Br^{-}$/MP (top) of obtained sliced data by one-dimensional processed at diagonal peak H22, diagonal peaks of 2D ROESY that mean irradiation positions for observing ROE correlations. For comparison, the $^{1}$H-NMR spectrum of 5 mM 14-6-14,2$Br^{-}$/MP is shown in the middle.

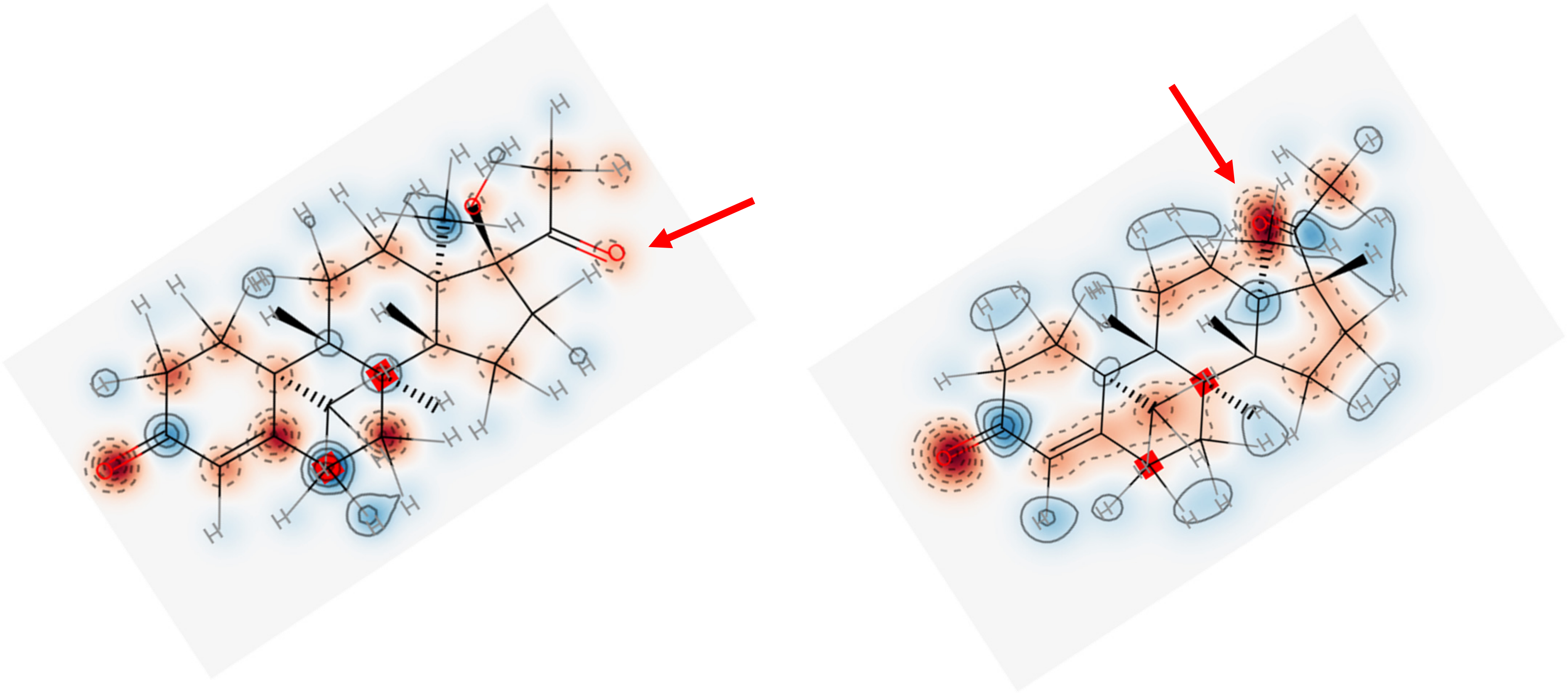

**Fig. S18** Electron density map of MP (left) and progesterone (right) calculated using Psi4 with the Mulliken charge scheme and the STO-3G basis set. The map visually represents the electron density of each atom in the progesterone molecule. Regions of high electron density are indicated in red, while regions of low electron density are shown in blue.
